# Spatial and Genomic Correlates of HIV-1 Integration Site Targeting

**DOI:** 10.3390/cells11040655

**Published:** 2022-02-14

**Authors:** Parmit Kumar Singh, Gregory J. Bedwell, Alan N. Engelman

**Affiliations:** 1 Department of Cancer Immunology and Virology, Dana-Farber Cancer Institute, Boston, MA 02215, USA; gregoryj_bedwell@dfci.harvard.edu; 2 Department of Medicine, Harvard Medical School, Boston, MA 02115, USA

**Keywords:** HIV/AIDS, retroviral integration, nuclear speckles, speckle-associated domains, lamina-associated domains, LEDGF/p75, CPSF6

## Abstract

HIV-1 integrase and capsid proteins interact with host proteins to direct preintegration complexes to active transcription units within gene-dense regions of chromosomes for viral DNA integration. Analyses of spatially-derived genomic DNA coordinates, such as nuclear speckle-associated domains, lamina-associated domains, super enhancers, and Spatial Position Inference of the Nuclear (SPIN) genome states, have further informed the mechanisms of HIV-1 integration targeting. Critically, however, these different types of genomic coordinates have not been systematically analyzed to synthesize a concise description of the regions of chromatin that HIV-1 prefers for integration. To address this informational gap, we have extensively correlated genomic DNA coordinates of HIV-1 integration targeting preferences. We demonstrate that nuclear speckle-associated and speckle-proximal chromatin are highly predictive markers of integration and that these regions account for known HIV biases for gene-dense regions, highly transcribed genes, as well as the mid-regions of gene bodies. In contrast to a prior report that intronless genes were poorly targeted for integration, we find that intronless genes in proximity to nuclear speckles are more highly targeted than are spatially-matched intron-containing genes. Our results additionally highlight the contributions of capsid and integrase interactions with respective CPSF6 and LEDGF/p75 host factors in these HIV-1 integration targeting preferences.

## 1. Introduction

Retroviral preintegration complexes (PICs) display variable preferences for genomic features associated with active versus repressive regions of chromatin during integration (for recent reviews, see [1,2]). The primate lentivirus HIV-1 in particular prefers to integrate into active transcription units/gene-dense regions of chromatin [3] in proximity to activating histone post-translational modifications such as histone H4 acetylation, histone H3K4 mono- and dimethylation (H3K4me1; me2), and histone H3K36 di- and trimethylation (H3K36me2; me3) [4,5,6,7]. Conversely, HIV-1 integration disfavors gene-sparse heterochromatic regions such as centromeric alphoid repeats [8] and lamina-associated domains (LADs) [9,10], as well as regions proximal to repressive epigenetic marks such as H3K9me2/3 and H3K27me3 [4,5,6]. Advances in 3D genomic mapping techniques such as tyramide signal amplification-sequencing (TSA-Seq) [11] and Spatial Position Inference of the Nuclear genome (SPIN) [12] have more recently informed the spatiality of HIV-1 integration targeting preferences [13,14,15]. Integration targeting preferences have been linked to speckle-associated domains (SPADs) [14,15] and Speckle and Interior Active 1 SPIN states [13] that typically localize distal from the nuclear envelope [11,12]. Conversely, chromatin regions associated with peripheral nuclear markers, such as LADs [9,10] and Near Lamina 1-2 and Lamina SPIN states [13], are strongly disfavored for HIV-1 integration.

Interactions of viral proteins with cognate cellular factors help to dictate HIV-1 integration targeting preferences. The interaction of integrase (IN), the viral factor whose catalytic function is required for integration, with cellular lens epithelium-derived growth factor (LEDGF)/p75 [16] targets integration into genes [17,18,19,20] and, more specifically, into the mid-regions of gene bodies [6,18,21]. Total levels of HIV-1 integration are suppressed through genetic ablation of LEDGF/p75 (LKO for LEDGF knockout) [18,19,22], suggesting that the stimulatory effect of LEDGF/p75 binding on HIV-1 IN activity in vitro [16,23] might be important for virus infection [13]. LEDGF/p75 binds diverse lentiviral IN proteins including those derived from primate and non-primate lentiviruses [24,25,26] and LEDGF/p75 accordingly directs the integration of diverse lentiviral species into gene mid-regions [15]. LEDGF/p75 can interact with pre-mRNA splicing factors [21] and alleviate the transcriptional block imposed by nucleosomes in vitro [27], suggesting that LEDGF/p75-dependent HIV-1 integration targeting might leverage cellular mRNA splicing and/or transcriptional elongation machineries.

In addition to the interaction between LEDGF/p75 and IN, the interaction between the HIV-1 capsid protein (CA) and cellular cleavage and polyadenylation specificity factor 6 (CPSF6) plays a role in primate lentiviral integration targeting [6,10,13,15,28]. The CA–CPSF6 interaction is important for nuclear penetrance of HIV-1 PICs [10,28,29,30,31]. Under baseline infection conditions, HIV-1 PICs colocalize with nuclear speckles [14], which correlates with genomic speckle and speckle-proximal integration targeting [13,14,15]. In the absence of the CA–CPSF6 interaction, HIV-1 fails to penetrate into the nuclear interior [10,28,29,31] and uncharacteristically targets lamina-associated chromatin for integration [10,13,15].

In the current study, we show that speckle and speckle-proximal regions strongly correlate with gene-dense regions on particular chromosomes, and that this correlation largely determines the pronounced HIV-1 integration targeting preferences for human chromosomes 16, 17, 19, and 22 [3,32]. While shared among primate lentiviruses including HIV-2 and simian immunodeficiency virus (SIV), non-primate lentiviruses did not preferentially target these human chromosomes. We additionally show that intronless genes in speckle and speckle-proximal regions are more highly targeted than are intron-containing genes in the same regions. Finally, we demonstrate that the known preference of HIV-1 to target gene mid-regions is primarily driven by integration in speckle and speckle-proximal regions of the genome. Altogether, our results demonstrate that speckle and speckle-proximal regions are the predominant target of HIV-1 integration and that integration into these regions largely accounts for the known integration targeting preferences for gene density and actively transcribed genes.

## 2. Materials and Methods

### 2.1. Datasets

Numerous activating histone post-translational modifications correlate with HIV-1 integration targeting [4,5,6,7]. Because H3K36me3 is also a preferred site of LEDGF/p75 binding to chromatin [27,33,34], we have analyzed it as a representative activating epigenetic mark. We have used chromatin immunoprecipitation-sequencing (ChIP-Seq) datasets for LEDGF/p75, H3K36me3, H3K27me3, and RNA polymerase II (Pol II) described in [27]. Genomic locations of SPADs were calculated from TSA-Seq data as described [11,14]. LAD coordinates were obtained directly from the authors [35]. SPIN states were downloaded from https://github.com/ma-compbio/SPIN (accessed on 18 January 2021) [12]. Please see Table S1 for genomic datasets and accession information that were used in this study. Table S2 reports the total number of genomic DNA fragments within these datasets as well as the average size and gene densities of the fragments.

Our analyses were based on human genome build hg19. Coordinates defined according to other genome builds were converted to hg19 coordinates using LiftOver (https://genome.ucsc.edu/cgi-bin/hgLiftOver (accessed on 18 January 2021)). Human RefSeq sequences, downloaded from the UCSC server [36], were demultiplexed as described [6] to convert independent isoforms such as splice variants of the same gene into single transcription units. The resulting nonredundant gene list, composed of 25,652 human genes, was used as the query dataset throughout this study unless indicated otherwise.

Sites of retroviral integration determined using ligation-mediated (LM)-PCR with genomic DNA isolated from infected cells were previously described [6,15,37,38,39]. These datasets included HIV-1 integration sites from human peripheral blood mononuclear cells (PBMCs) [39]. HIV-1 integration sites were also derived from an isogenic set of HEK293T cells including wild-type (WT), LEDGF/p75 knockout (LKO), CPSF6 knockout (CKO), and cells knocked out for both LEDGF/p75 and CPSF6 (DKO for double knockout) [6]. HIV-2, equine infectious anemia virus (EIAV), bovine immunodeficiency virus (BIV), and feline immunodeficiency virus (FIV) integration sites were also analyzed from human HEK293T cells [15], while SIV integration sites were from human PBMCs [39]. Integration sites from WT or LKO Jurkat T cells, and from WT Jurkat T cells infected with HIV-1 harboring either the N74D or A77V CA mutation, were from Li et al. [15]. In silico-generated random integration control (RIC) datasets made to mimic wet bench-based LM-PCR techniques were constructed as previously described [15]. Random sites to control for HIV-1/SIV integration targeting frequencies in PBMCs were from [39]. All retroviral integration site datasets used in this work are listed in Table S1.

### 2.2. ChIP-Seq Analyses

ChIP-Seq reads were processed and aligned as per the description from the published method [27]. In short, sequences were aligned to hg19 using Bowtie2 [40]. Sam outputs were converted to bam files with SAMtools [41]. Bam files were subsequently sorted using Picard Tools (http://broadinstitute.github.io/picard/ (accessed on 18 January 2021)) and peak calling was done using macs2 v2.1.1.20160309 with sorted bam files as input (macs2 callpeak -t $1 -c $2 --broad -g 2.7e9 --broad-cutoff 0.01 -n $3 -f BAM -B) [42]. Assigned peak thresholds were adjusted based on the following ChIP-Seq control datasets: SRR7525652 (input-native) for LEDGF/p75 (SRR7525645) and SRR7525651 (input) for Pol II (SRR7525644), H3K36me3 (SRR7525649), and H3K27me3 (SRR7525650) (Table S1).

### 2.3. Spatial Position Inference of the Nuclear Genome (SPIN) States

Coordinates corresponding to the 10 SPIN states were obtained as described above [12]. Coordinates were converted to hg19 coordinates using liftover (https://genome.ucsc.edu/cgi-bin/hgLiftOver (accessed on 18 January 2021)) and the hg38ToHg19.over.chain file. The coordinate conversion retained 4298 of 4510 originally reported regions. Table S2 reports the total number of genomic DNA fragments per SPIN state, as well as the average size of these fragments.

### 2.4. Random Sites

Random datasets (not including the random integration control datasets) were constructed by BEDtools [43] to approximate the average fragment lengths of genomic DNA fragments in comparator datasets. For example, the average fragment lengths in the H3K36me3 and H3K27me3 ChIP-Seq datasets were 4.67 and 4.62 kb, respectively (Table S2). Therefore, random datasets that contained 4.7 kb fragments were constructed and analyzed for comparison. Additional random datasets harboring 860, 532, and 100 bp fragments were constructed to similarly mirror experimental Pol II ChIP-Seq, LEDGF/p75 ChIP-Seq, and SPAD datasets, respectively (Table S2). For each experimental method, 5 independent random datasets containing at least 1,000,000 length-matched fragments were generated, giving a total of 20 random datasets (Figure S1 and Table S2).

### 2.5. Gene Density Calculations

We calculated average gene density per Mb (±500 kb) from midpoint genomic regions, defined as the central or middle nucleotide between the start and the end positions of analyzed genomic fragments (e.g., SPADs or ChIP-Seq fragments). Within each fragment, genes were parsed to remove overlapping transcription units to maintain consistency with prior gene density analyses [6,44].

### 2.6. Analysis of HIV-1 Integration Sites with Global Genomic Annotations

Percent integration in genes was quantified using BEDtools [43]. To account for gene size, we normalized percent genic HIV-1 integration according to two methods: (1) by percent genic RIC sites across all genes and (2) by dividing percent genic integration by the total length of all genes. Both methods produced similar results and we compared these methods to previous methodology that reported that intronless genes were disfavored targets for HIV-1 integration [21].

### 2.7. Length-Wise Analysis across Genes

To analyze integration profiles across genes, each gene was divided into ten equal segments and expressed as percent of total gene length. The percent of HIV-1 integration was subsequently calculated for each segment with respect to the total number of integration sites in a given dataset.

### 2.8. Identification of Region-Associated and Non-Associated Genes

To define genes associated with analyzed genomic regions, BEDTools was used to identify all genes that overlapped with ChIP-Seq, SPAD, and SPIN state annotations. Genes with overlapping coordinates were considered as associated whereas non-overlapping genes were considered as non-associated genes [43]. Overlapping regions between SPIN states and SPADs were similarly identified [43].

### 2.9. Gene Expression per Chromosome

To quantify the average gene expression per chromosome, we used RNA-seq data derived from naïve CD4 T cells (https://www.proteinatlas.org/about/download; accessed on 13 July 2021) [45]. The average gene expression per chromosome was calculated as the total expression of all expressed genes divided by total chromosome length.

### 2.10. Statistical Tests

Fisher’s exact and chi-square tests were used to calculate differences. Resulting *p* values and methodologies are indicated in Supplemental Tables and/or figure legends. Data Analysis (Regression) in Excel was used to address variabilities in genic integration targeting as a function of intron content.

## 3. Results

### 3.1. Experimental Strategy

In this study, we have sought to define the relationships between genomic and spatial correlates of HIV-1 integration targeting. To do this, we have performed meta-analyses using various datasets corresponding to both spatial and genomic markers associated with HIV-1 integration, and then intersected these findings with previously mapped integration sites. To address the potential roles of integration targeting cofactors LEDGF/p75 and CPSF6 in the resulting phenotypes, we analyzed integration site datasets derived from factor-depleted cells or from cells infected with CA mutant viruses that are defective for CPSF6 binding [6,15].

Owing to the different techniques that were previously used to generate genomic DNA datasets, the annotated regions within them varied in size. SPAD and LAD datasets, which were previously determined using TSA-Seq [11] and Dam-ID [35] methodologies, respectively, harbored comparatively large numbers (0.9–1.5 million) of relatively small (≤0.1 kb) annotated regions (Tables S1 and S2). SPIN state fragments, by contrast, encompassed much fewer DNA segments of comparatively large size (~0.5–1.4 Mb). The ChIP-Seq datasets (Table S1) utilized here, which included data for LEDGF/p75, Pol II, H3K36me3, and H3K27me3, each contained more than 10^4^ annotations that ranged in size from approximately 0.5 to 5 kb (Table S2).

To account for the different sizes of DNA fragments and to ensure that the size differences themselves would not significantly bias our comparisons, we initially assessed the gene densities associated with different series of computationally-generated random DNA fragments. Four different sizes, which ranged from 0.1 to 4.7 kb to mimic the average fragment sizes present in the SPAD/LAD and ChIP-Seq datasets (Table S2), were assessed for gene density composition. For each fragment size, 5 random datasets harboring either one million (532 bp, 860 bp and 4.7 kb) or 1,547,458 fragments, which was chosen to precisely match the 100 bp-fragment-containing SPAD dataset (Table S2), were generated, yielding size-matched totals of 5 million or 7,737,290 random fragments per genomic annotation. The gene density profiles of these in silico-generated DNA fragments appeared largely similar to one another and independent of query fragment size (Figure S1). The average gene density of the human genome was therefore defined as the average density of all 22,737,290 random fragments, or 8.66 genes/Mb. For the sake of this study, we considered segments that map to regions that harbor more than 9 gene/Mb as gene-dense and segments that map to regions of ≤9 genes/Mb as residing in gene-poor regions of the human genome.

### 3.2. Gene Density Profiles of Genomic and Spatial Annotations

#### 3.2.1. Gene Density Profiles of SPADs and LADs

**Figure 1:**
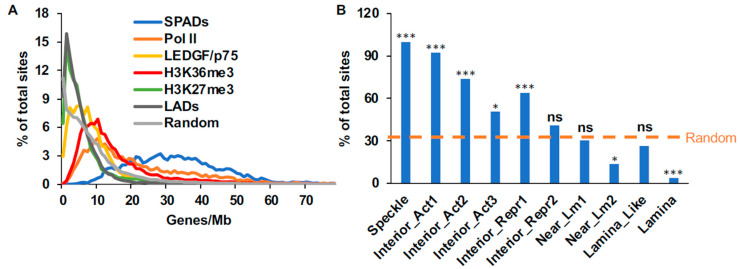
Distributions of human genome annotations with respect to gene density. (**A**) Average gene density (X-axis), which was calculated for 1 Mb genomic DNA regions, were plotted against the % of sites having the same gene density (Y-axis). The computationally-generated curve from Figure S1P was replotted as a representative random control. (**B**) Proportions of SPIN state fragments with gene-densities > 9 genes/Mb. The random 32.1% value (Table S2) is indicated by the horizontal dashed line. Asterisks indicate *p* values versus random as determined by Fisher’s exact test: ***, *p* < 0.0001; *, *p* ≤ 0.01; ns, not significant (Table S3).

Although HIV-1 integration is known to greatly favor gene-dense chromosomal regions and SPADs [3,14,15], the correlation of these two targeting metrics in the human genome has not been directly assessed. We accordingly calculated the gene density at the midpoint of each SPAD fragment as described in Materials and Methods. Our results revealed an approximately symmetrical distribution with an average gene-density of 32.6 genes/Mb for all SPAD fragments (Figure 1A). In total, 98.5% of SPAD fragments mapped to gene-rich regions of the human genome, which was significantly enriched *(p* < 10^−5^) compared to the random 32.1% value (Tables S2 and S3). Contrary to SPADs, LADs generally reside in gene-sparse regions [46] and are typically avoided during HIV-1 integration [9,10]. In accordance with previous data, we found that most LAD annotations (87%) mapped to gene-sparse regions of human DNA, which was also enriched *(p* = 0.002) compared to random (Tables S2 and S3 and Figure 1A).

#### 3.2.2. Gene Density and SPAD Profiles of SPIN States

Wang and colleagues recently expanded the scope of SPAD and LAD annotations by defining ten distinct spatial compartments within the human genome, which they refer to as Spatial Position Inference of the Nuclear genome (SPIN) states [12]. The 10 SPIN states radiate approximately outwardly within a theoretical nucleus from Speckle to Lamina. In this way, the “order” of the 10 states from the innermost state to the outermost state is: Speckle, Interior Active 1, Interior Active 2, Interior Active 3, Interior Repressive 1, Interior Repressive 2, Near Lamina 1, Near Lamina 2, Lamina-like, and Lamina. As expected, the average gene density of Speckle regions, 30.6 genes/Mb, was similar to that of SPADs and was the highest among all the SPIN states (Table S2). The average gene density of the remaining SPIN states decreased outwardly from Speckle regions (Figure S2A–I and Table S2). To better assess how the different SPIN states correlated with gene-dense regions of the genome, we calculated the percentage of each SPIN state that resided in regions with gene densities greater than random (9 genes/Mb). The result (Figure 1B) demonstrated that the percentage of each SPIN state that resides in regions with gene density greater-than random decreases approximately linearly as one moves outward from the Speckle state to Lamina regions. Two notable exceptions to this overall trend were SPIN states Interior Active 3 and Near Lamina 2, which overlapped with fewer gene-dense regions than expected based on neighboring SPIN states (Figure 1B). Two of the three outermost SPIN states, Near Lamina 2 and Lamina, significantly overlapped with gene-sparse regions of the human genome (Figure 1B and Table S3).

The granularity inherent to SPIN state classifications allowed us to further refine the relationship between gene density and nuclear speckles. As expected, the vast majority (83%) of SPADs overlapped with Speckle regions (Table S4). An additional 14.9% of SPADs overlapped with Interior Active 1 regions. Notably, despite the fact that Interior Active 2, Interior Active 3, and Interior Repressive 1 regions were enriched for gene-dense chromatin (Figure 1B), they harbored in total just 0.3% of SPADs. Thus, while Speckle and speckle-proximal chromatin correlated strongly with gene-dense chromatin, not all gene-dense chromatin is similarly associated with nuclear speckles.

#### 3.2.3. Gene Density Profiles of ChIP-Seq Sequences

We next determined the gene density profiles of genomic DNA annotations associated with HIV-1 integration targeting including H3K36me3, Pol II, and LEDGF/p75, as well as the anti-correlative marker H3K27me3 [4,5,27]. Regions of Pol II occupancy were significantly enriched for gene density compared to random (*p* < 10^−5^), with 76.8% of Pol II fragments mapping to gene-dense regions (Figure 1A, Tables S2 and S3). The majority of H3K36me3 peaks (62%; *p* < 10^−5^) similarly mapped to gene-dense regions of chromatin (Figure 1A, Tables S2 and S3). Although LEDGF/p75-associated fragments with average density of 10 genes/Mb were enriched compared to random (Figure 1A), the overall gene density profile of LEDGF/p75-associated fragments, 8 genes/Mb, was statistically indistinguishable from random (Tables S2 and S3). H3K27me3 association by contrast trended more strongly toward gene-sparse regions *(p* = 0.03), with just 17.8% of these fragments residing in gene-dense regions (Figure 1A, Tables S2 and S3).

### 3.3. Chromosomal Distributions of Genomic and Spatial Annotations

**Figure 2:**
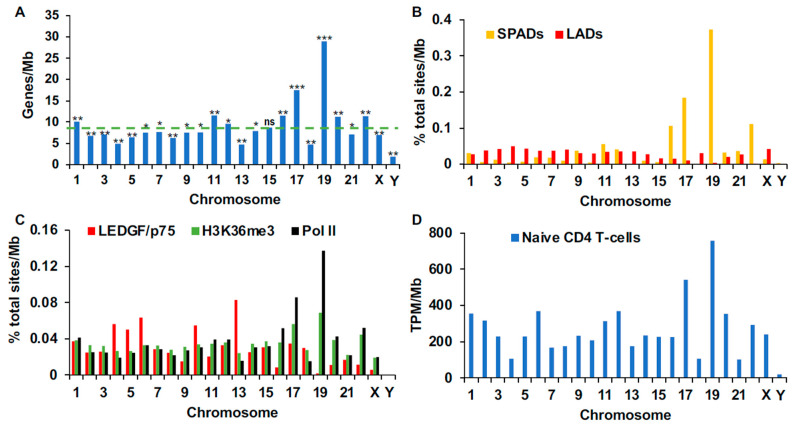
Chromosomal-level analytics. (**A**) Gene densities of human chromosomes. The average density of the human genome, 8.7 genes/Mb, is indicated by green horizontal line. (**B**) Chromosomal distributions of SPAD and LAD sites per Mb. (**C**) Distributions of LEDGF/p75-, H3K36me3-, and Pol ll-associated sites per Mb. (**D**) Total transcripts per million (TPM) per chromosome, normalized by chromosome length. Panel **A** *p* values: *, ≤0.005; **, <10^−10^; ***, <10^−170^; ns, not significant (Table S5).

Human chromosomes can be categorized as gene dense or gene poor based on their inherent gene densities [47]. Our calculations revealed chromosomes 19 and 17, with average gene densities of 29 and 17.5 genes/Mb, respectively, as most highly enriched compared to random (Figure 2A and Table S5). The next level of gene enrichment, which encompassed similar densities of 11.2 to 11.6 genes/Mb, mapped to chromosomes 11, 16, 20, and 22. Chromosomes 4, 13, and 18 were the most gene-sparse autosomal chromosomes (Figure 2A and Table S5).

Given that the genomic and spatial annotations we analyzed thus far defined relationships with respect to gene density, we hypothesized that these relationships should also manifest at the chromosomal level. To test this hypothesis, we quantified the percentages of SPADs, LADs, and ChIP-Seq sites for each chromosome, normalized these values for total chromosome length, and then assessed these findings per human chromosome as well as the genome as a whole (Tables S6 and S7). While SPADs, Pol II, and H3K36me3 association regions highly correlated with gene density across chromosomes, LADs and LEDGF/p75-associated regions negatively correlated, though the difference for LEDGF/p75-bound sites was not significant (*p* = 0.13; Table S6). At the level of individual chromosomes, SPADs and Pol II-associated regions were highly enriched on gene-dense chromosomes 16, 17, 19, and 22 (Figure 2B,C; Table S7). H3K36me3 was additionally enriched on these chromosomes, though its enrichment on chromosomes 16, 17, and 19 appeared less pronounced than Pol II and SPADs (Figure 2; Table S7). By contrast, LADs and sites of chromatin-bound LEDGF/p75 in general partitioned to gene-sparse chromosomes (Figure 2A–C; Tables S5 and S7).

We next analyzed chromosomal partitioning of human SPIN states. While Speckle and Interior Active 1 states correlated strongly with gene density, Interior Repressive 1, Near Lamina 1 and 2, and Lamina SPIN states negatively correlated (Table S6). At the level of individual chromosomes, 16, 17, 19 and 22 were enriched in Speckle and Interior Active 1 regions (Figure S3A,B). Chromosomes other than 16, 17, 19, and 22 were generally more enriched for Lamina and lamina-proximal SPIN states (Figure S3A,C,E). Notably, while gene density did partially correlate with the observed differences in feature occupancy per chromosome, other chromosomal biases were also apparent. For example, despite the fact that chromosomes 11, 16, 20, and 22 harbored comparable gene densities (11.6, 11.5, 11.2, and 11.4 genes/Mb, respectively), there were marked differences in Speckle and speckle-associated chromatin occupancy between them (Figure 2A,B and Figure S3A,B). This observation is consistent with our assertion that not all gene-dense chromatin lies similarly proximal to nuclear speckles. Size-matched random controls were seen to distribute similarly across all human chromosomes (Figure S4).

In addition to the genomic and spatial correlates described above, active transcription is a known correlate of HIV-1 integration targeting [3], and, at the genome level, gene expression strongly correlated with gene density (Table S6; *p* < 10^−5^). While the most gene-dense chromosomes (chromosomes 17 and 19) also showed the highest level of overall transcriptional activity (total TPM/Mb), other SPAD-enriched chromosomes, for example 16 and 22, showed less expression than chromosomes such as 2 and 6, which were comparatively gene-sparse and enriched for LADs and LEDGF/p75 occupancy (Figure 2). Thus, the overall transcriptional activity of a given chromosome did not necessarily correlate with SPAD enrichment.

### 3.4. Chromosomal Distributions of Retroviral Integration Sites

We have thus far analyzed chromosomal biases of positive and negative correlates of HIV-1 integration and the associations between them. To better understand the impact of these respective biases on HIV-1 integration targeting, we next analyzed integration sites recovered from genomic DNA following infection of three different cell types: human PBMCs, HEK293T, and Jurkat T cells. PBMCs were derived from human blood donors and accordingly harbored primary CD4+ T cells. While not targets of clinical infection, HEK293T and Jurkat T cells were nevertheless chosen due to accessibility of complementary datasets derived from LEDGF/p75- and CPSF6-depleted cells, or from cells infected with CA mutant viruses that are defective for CPSF6 binding [6,13,15].

As expected based on prior work [13,32,48], chromosomal patterns of HIV-1 integration were largely similar across cell types. Additionally, consistent with previous results [3,32], we found that integration was most strongly biased towards chromosomes 16, 17, 19, and 22 (Figure S5A,F–H). These findings noticeably mirrored the observed biases for speckle-associated chromatin (Figure 2B and Figure S3A,B). Notably, despite having similar gene densities, gene expression levels, and occupancies of Pol II and H3K36me3, chromosome 20 was less targeted for integration than chromosomes 16 and 22 (Figure 1 and Figure S5A), which is consistent with the notion that speckle-associated chromatin is a dominant correlate of HIV-1 integration targeting.

Previous results have indicated that HIV-1 integration targeting preferences are governed largely by interactions between viral CA and IN with cellular CPSF6 and LEDGF/p75, respectively [6,10,14,15,21]. Crucially, however, ablation of these respective interactions affected integration targeting in distinct ways. In the absence of the CA–CPSF6 interaction, integration targeting favored LADs and other gene-sparse chromatin regions, disfavored integration into genes relative to integration in the presence of CPSF6 and lacked clear positional biases within genes when they were targeted for integration [6,10]. Disruption of the IN-LEDGF/p75 interaction showed some of these same effects, though the phenotypes were generally less extreme [6,10,13,15]. In addition, disruption of the IN-LEDGF/p75 interaction redirected HIV-1 integration from mid-regions of gene bodies toward gene 5′ ends [6,21].

We have analyzed the roles of LEDGF/p75 and CPSF6 in primate and non-primate lentiviral integration targeting at the chromosome level using knockout cells or from infections initiated with CA mutant viruses. Although we previously knocked out *CPSF6* from WT and LKO HEK293T cells, creating respective CKO and double knockout DKO cells [6], we have been unable to similarly knockout *CPSF6* from T cell lines commonly used in HIV-1 infection experiments. We accordingly analyzed integration sites from Jurkat T cells infected with CA mutant viruses N74D and A77V [15], each of which is defective for CPSF6 binding [49,50]. In contrast to CPSF6, integration sites were available for both HEK293T and Jurkat T LKO cells [6,15].

Consistent with previous reports [6,10], loss of CPSF6 imparted dramatic effects on chromosomal biases of HIV-1 integration targeting. The chromosomes with the largest percentages of integration in CKO cells were 3, 4, 6, and 13, while integration in LKO cells was still predominantly targeted to chromosomes 16, 17, 19, and 22 (Figure S5A and Table S8). Similar results were observed in LKO Jurkat cells as well as in WT Jurkat cells infected with N74D and A77V mutant viruses (Figure S5G,H). LAD-enriched autosomal chromosomes were generally more highly targeted in CKO cells than in WT cells (Figure 1B, Figure S5A,H). LAD-enriched chromosomes additionally exhibited slightly elevated targeting frequencies in LKO cells, but this phenotype was noticeably less than the levels of integration enrichment in chromosomes 16, 17, 19, and 22 (Figure S5A,G).

We previously demonstrated that non-primate lentiviruses display minimal-to-no preferences for integrating into SPADs [15]. We hypothesized that because these viruses were not biased towards SPADs, they would similarly lack strong enrichment towards SPAD-rich chromosomes. Indeed, non-primate lentiviral EIAV, BIV and FIV integration lacked the preference for SPAD-rich chromosomes 16,17, 19 and 22 that was observed for HIV-1 (Figure S5). Primate lentiviruses HIV-2 and SIV, by contrast, demonstrated integration targeting preferences similar to those observed for HIV-1 (Figure S5A,B,F). HIV-1 and HIV-2 integration preferences in double knockout DKO cells were more similar to those observed in CKO as compared to LKO cells, indicating that the CA–CPSF6 interaction predominately determines chromosomal levels of primate lentiviral integration site targeting [6].

### 3.5. Genic Targets of HIV-1 Integration

HIV-1 integration is heavily biased towards genes, with ~75–85% of all integration events taking place within transcription units [3,4,6,51]. In addition, specific genes have been found to be targeted more often than others, suggesting that particular features are “sought out” by the mechanism of genic integration targeting [9,13,14,52]. What these particular features are, however, and how they might work together, remains largely unknown, though general features of active transcription, high intron density, and proximity to nuclear speckles have been associated with genic HIV-1 integration targeting [3,6,13,21].

**Figure 3:**
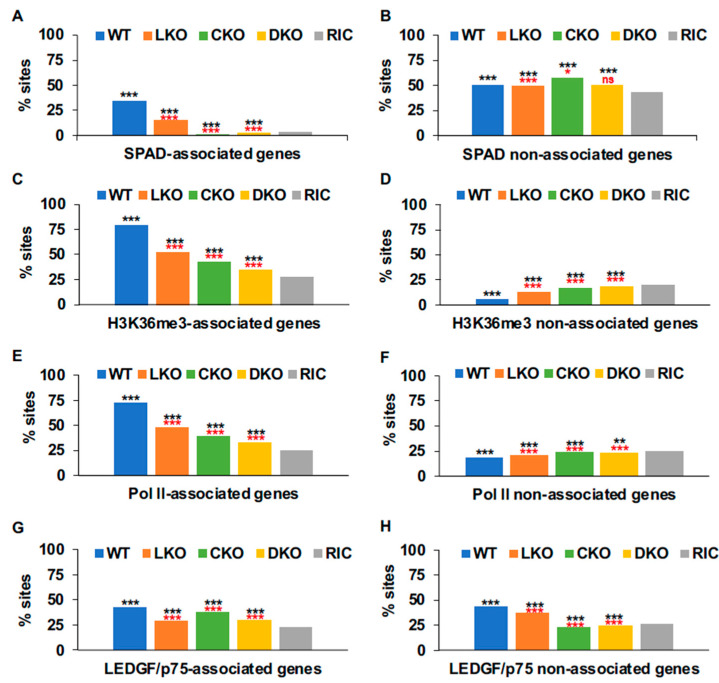
Integration targeting frequencies into different gene sets. (**A**,**B**) HIV-1 integration into SPAD-associated (panel **A**) and non-associated (panel **B**) genes for the indicated HEK293T cell type. (**C**,**D**) Integration into H3K36me3-associated (panel **C**) and non-associated (panel **D**) genes. (**E**,**F**) Integration into Pol II-associated (panel **E**) and non-associated (panel **F**) genes. (**G**,**H**) Integration into LEDGF/p75-associated (panel **G**) and non-associated (panel **H**) genes. RIC, random integration control. *p* values, calculated using Fisher’s exact test, show differences versus RIC in black and versus WT HEK293T cells in red. ***, *p* < 0.001; **, *p* ≤ 0.01; *, *p* < 0.05; ns, not significant (*p* > 0.05). See Table S9 for comprehensive listing of *p* values.

Primate lentiviral DNA integration is biased towards chromosomes 16, 17, 19, and 22 (Figure S5A,B,F) and these chromosomes are enriched in SPADs, Pol II occupancy, and H3K36me3 occupancy (Figure 2). To understand the relationship more fully between these individual markers and HIV-1 integration, we divided each set of genomic DNA fragments into two subpopulations based on whether genes associated with the marker under study. We then plotted integration frequencies into these marker-associated versus marker-non-associated genes. Between 33% and 42% of HIV-1 integration occurred in SPAD-associated genes across cell types. Although these values were lower compared to the frequencies of integration into H3K36me3- and Pol II-associated genes, SPAD-associated genic integration was most highly enriched due to the low frequency of these annotations in the human genome, which, depending on computational methodology, was 3.3% [15] or 5.3% [39] (Figure 3A, Figures S6A, S7A and S8A). Thus, whereas integration into SPAD-associated genes was enriched 8 to 10-fold compared to random across cell types, integrations into H3K36me3- and Pol II-associated genes were enriched approximately 3-fold. Integration targeting frequencies of LEDGF/p75-associated genes were still closer to random, showing approximate 1.5 to 2-fold enrichments across cell type (Figure 3 and Figures S6–S8; see Tables S9–S12 for statistical summaries across different integration datasets and cell types).

We also assessed integration site targeting frequencies of the reciprocal marker-non-associated gene populations. Integration into genes that did not associate with SPADs was comparatively close to random, displaying mere approximate 10% to 20% enrichments across cell type (Figure 3B, Figures S6B, S7B and S8B). Genes that did not associate with H3K36me3 or Pol II were disfavored for HIV-1 integration independent of cell type (Figure 3D,F, Figures S6D,F, S7D,F and S8D,F, Tables S9–S12).

#### 3.5.1. Integration as a Function of Intron Content

HIV-1 integration occurs preferentially in intron-rich genes [6,21]. It was therefore of interest to assess how the preference for intron density correlated with SPAD, Pol II, H3K36me3, and LEDGF/p75 genomic regions. In accordance with a previous study [21], we employed coordinates for total RefSeq transcripts in this analysis, rather than the demultiplexed gene set that we routinely use for integration site mapping studies. We calculated an average intron density of 0.15 per kb from a set of 61,178 transcripts (Table S13). Transcripts that overlapped with speckle-associated regions, including SPADs and the Speckle SPIN state, were intron dense (Table S13). Conversely, transcripts associated with LEDGF/p75 binding sites were intron sparse, while LEDGF/p75 non-associated transcripts were marginally intron-dense (Table S13; *p* = 0.02). In general, the intron densities of transcripts associated with each of the 10 SPIN states decreased linearly moving outward from the interior region of the nucleus with the notable exception of Lamina-like, which harbored transcripts with greater intron density compared to those associated with several inner neighbor states (Table S13).

**Figure 4:**
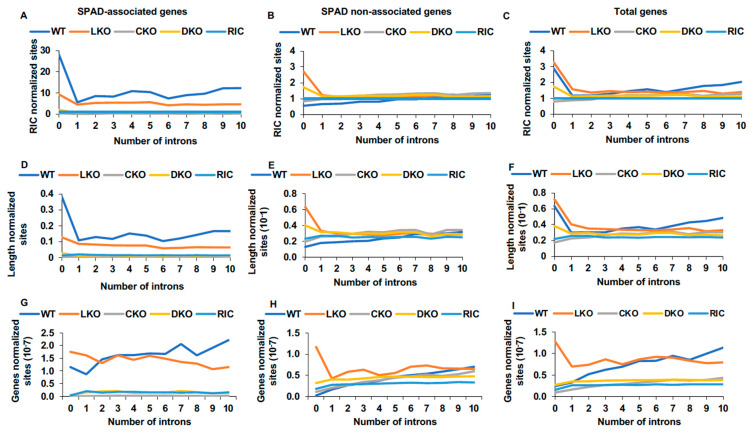
Speckle-associated intronless genes are highly targeted by HIV-1 for integration. (**A**) Integration targeting frequency in SPAD-associated genes in HEK293T cells as a function of intron content. Results are normalized to random integration control (RIC). (**B**,**C**) Same as in panel **A**, except for SPAD non-associated genes (panel **B**) and total genes (panel C). (**D**–**F**) Same as in panels **A**–**C**, except that results were normalized by total gene length in Mb. (**G**–**I**) The same gene sets as above were analyzed as previously described [21] where the average integration (%) for each intron group was calculated.

It has been reported that despite comparatively high levels of expression, intronless genes are targeted for integration less frequently than are intron-containing genes [21]. Overall, just 19% of intronless genes reside in speckle-associated chromatin. Because integration is highly biased towards speckle-associated chromatin, we assessed the level of integration within speckle-associated intronless genes. For this analysis, we grouped SPAD-associated and non-associated transcripts according to the number of introns (up to 10 introns) and then quantified percent integration into each associated genomic DNA fragment. After normalization to random genic sites or to the total length of transcripts in a given intron group, we found that SPAD-associated intronless genes were significantly more enriched for integration targeting relative to genes that contained one intron across cell types (Figure 4A,D, Figure S9A,D,G and Table S14). Moreover, in HEK293T cells and PBMCs, integration into SPAD-associated intronless genes was favored over the vast majority of intron-containing transcripts and genes that were analyzed. The significance of this phenotype was highlighted by comparing integration targeting frequencies in SPAD-non-associated genes versus total genic populations (SPAD-associated + non-associated genes). While integration in intronless genes was disfavored for SPAD-non-associated genes, it was observed across all genes (Figure 4C,F, Figure S9C,F,I and Table S14).

This finding stands in contrast to the previous report that intronless genes were the least targeted genes amongst all human genes [21]. To address this discrepancy, we repeated the analysis as per the method described in Singh et al., which reproduced the published findings (Figure 4G–I and Figure S10). The reason for the apparent difference in findings is that our current analysis accounted for random integration targeting frequency whereas the original analysis lacked this normalization component [21]. Because intronless genes are comparatively short relative to intron-containing genes and less abundant, it is unsurprising that they would be integrated into less frequently than intron-containing genes. However, relative to the extent of random integration, it is clear that intronless genes in speckle-associated regions are targeted more frequently than expected.

#### 3.5.2. Host Factor Roles in Genic Integration Site Targeting

Consistent with our previous analysis of bulk genic integration targeting frequency [6], HIV-1 integration in LKO, CKO, and DKO HEK293T cells increasingly lost the bias towards SPAD-associated, Pol II-associated, and H3K36me3-associated genes that were observed in WT cells (Figure 3A,C,E). These same trends were observed in Jurkat LKO cells as well as in Jurkat cells infected with CPSF6-defective N74D and A77V mutant viruses (Figures S6 and S7, Tables S10 and S11). Concomitant changes in respective non-associated genes were not observed. In fact, independent of cell type, lack of effective host factor binding enhanced integration into Pol II- and H3K36me3-non-associated genes (Figure 3D,F, Figures S6D,F and S7D,F). LKO reduced integration into LEDGF/p75-associated genes by about 13% in both HEK293T and Jurkat T cells (Figure 3G and Figure S6G). Integration into LEDGF/p75-non-associated genes was similarly reduced in Jurkat LKO cells (Figure S6H).

The preference for HIV-1 to target SPAD-associated intronless genes for integration was noticeably diminished in HEK293T and Jurkat LKO cells (Figure 4A,D, Figure S9G and Table S14). Conversely, SPAD-non-associated intronless genes were preferentially targeted in HEK293T LKO cells (Figure 4B,E and Table S14). HIV-1 integration also favored SPAD-non-associated intronless genes in Jurkat LKO over Jurkat WT cells, though the targeting frequency in LKO cells did not differ significantly from random (Figure S9G,H and Table S14). In contrast to SPAD-associated genes, intron-containing SPAD-non-associated genes showed a more apparent correlation between the number of introns and the level of integration targeting, similar to what was reported previously for all genes (Figure 4B,E, Figure S9B,E,H and Table S15) [6,21]. For SPAD-non-associated genes, we determined in WT and LKO cells adjusted R^2^ values of ≥0.81 and ≤0.23, respectively, for the correlation of intron number and integration targeting frequency (Table S15). Omitting intronless genes from SPAD-associated and all gene comparisons moreover improved adjusted R^2^ values in the majority of the WT cell types.

#### 3.5.3. Integration in Gene-Dense Regions

**Figure 5:**
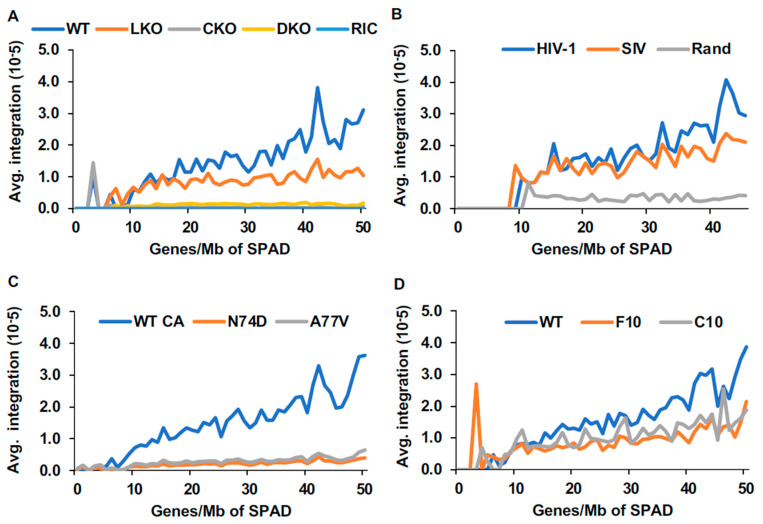
HIV-1 preferentially integrates into high gene-dense regions of SPADs. For each gene density group (X-axis), average viral integration frequency was plotted on Y-axis. (**A**) HIV-1 integration sites from WT, LKO, CKO, and DKO HEK293T cells. (**B**) Integration sites from HIV-1 or SIV-infected PBMCs. (**C**) Integration sites from Jurkat T cells infected with indicated CA virus (WT or mutant). (**D**) Integration sites from WT and LKO Jurkat T cells. The random integration control (RIC) plotted in A applies to panels **C** and **D** as well. Rand, random control sites from PBMC study.

HIV-1 integration has long been associated with gene-dense chromatin [3]. However, our analysis to this point suggested that gene density alone is insufficient to fully account for integration targeting preferences. Moreover, the correlation with SPADs and speckle-associated chromatin [13,14] (Figure 1 and Figure S5) appeared to be among the strongest predictors of integration site selection. To further address the roles played by gene density versus speckle-associated chromatin regions, we calculated integration frequencies in SPAD regions that were stratified according to average gene density. The results presented in Figure 5 revealed a strong positive correlation between percent integration and gene density within SPADs across cell types, indicating that gene-dense SPADs are the most highly targeted SPADs for HIV-1 integration. While the correlation persisted though was less pronounced in LKO cells, it was expectedly negated by CKO or via infection with N74D and A77V viruses [6,10,14,15] (Figure 5).

**Figure 6:**
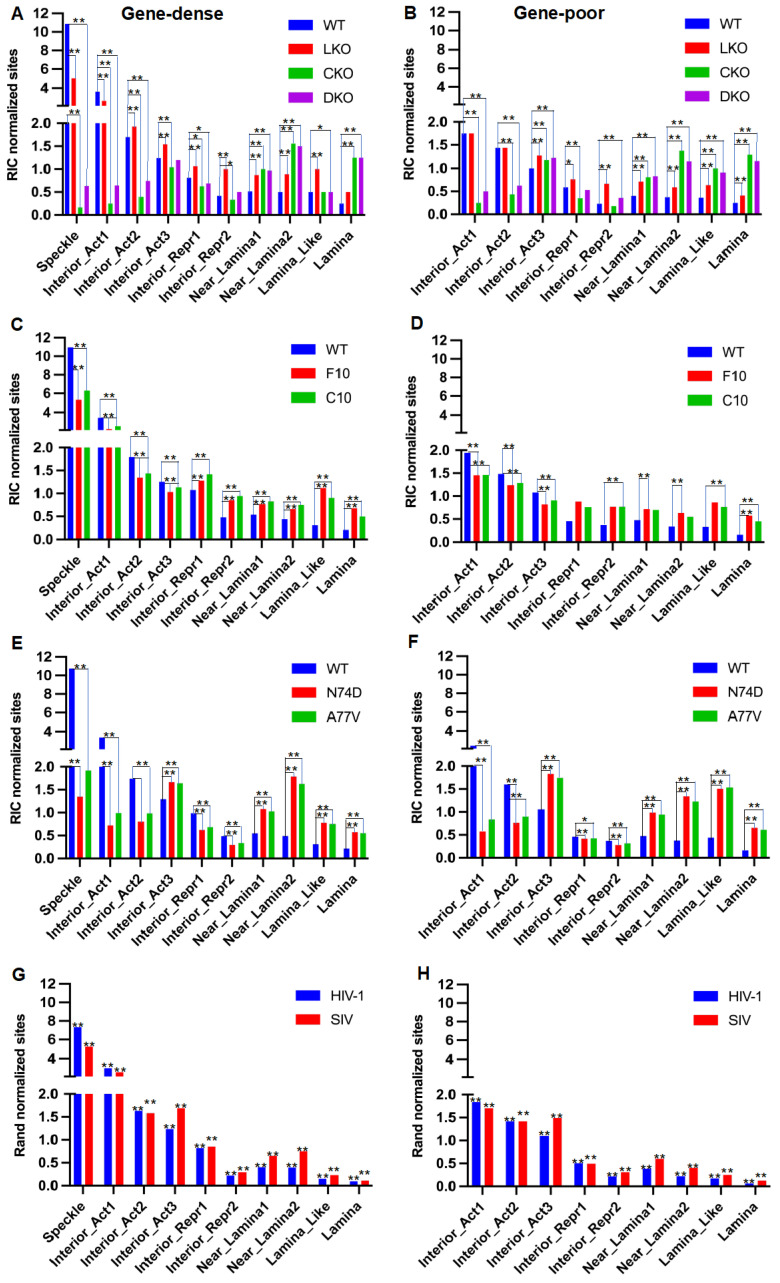
Integration targeting frequencies in different SPIN states as a function of gene density. (**A**,**B**) Integration targeting frequencies in the indicated SPIN states in WT and KO HEK293T cells. The average gene density of each SPIN state site was stratified as >9 (panel **A**) versus ≤9 (panel **B**). (**C**,**D**) Same as in panels **A** and **B**, except that integration sites were from Jurkat WT and LKO (F10 and C10) cells. (**E**,**F**) Same as in panels **A** and **B**, except that integration sites were obtained from Jurkat cells infected with WT or indicated capsid mutant virus. (**G**,**H**) Same as in panels **A** and **B**, except that integration sites were obtained for HIV-1 and SIV in PBMCs. Integration sites were normalized with respect to computationally-matched random controls RIC (panels **A**–**F**) and Rand (panels **G**,**H**). *p* values, calculated using Fisher’s exact test, show differences versus WT (**A**–**F**) and verses Rand (**G**,**H**). **, *p* < 0.0001; *, *p* < 0.05. See Table S16 for comprehensive listing of *p* values.

Integration targeting of gene-dense chromosomal regions is suppressed in CKO cells or in cells infected with CPSF6 binding-defective viruses [6,50] (Figure S5). Moreover, under these conditions, HIV-1 fails to penetrate into interior regions of cell nuclei [10,28,29,31]. It was therefore of interest to assess the influence of gene density in HIV-1 integration targeting across the different SPIN states. We accordingly divided each SPIN state into two groups: those with gene densities > 9/Mb (greater than random) and those with gene densities of ≤ 9/Mb (less than or equal to random). As expected, integration was most favored in the highly gene-dense Speckle SPIN state independent of cell type. Moreover, independent of cell type, integration targeting frequency decreased as one moved from the nuclear exterior out toward the peripheral SPIN states (Figure 6). Integration was favored in SPIN states peripheral to Interior Active 2 in LKO cells as compared to matched WT cells (Figure 6A–D; see Table S16 for comprehensive statistics). In HEK293T CKO cells and in Jurkat T cells infected with N74D and A77V capsid mutant viruses, HIV-1 integration targeting shifted more dramatically outward and into the peripheral SPIN states. Under these conditions, the extents of integration targeting in both gene-dense and gene-poor regions of chromosomal DNA were largely similar to one another (Figure 6A,B,E,F).

#### 3.5.4. Speckle-Associated Genes Primarily Bias HIV-1 Integration into Gene Mid-Regions

**Figure 7:**
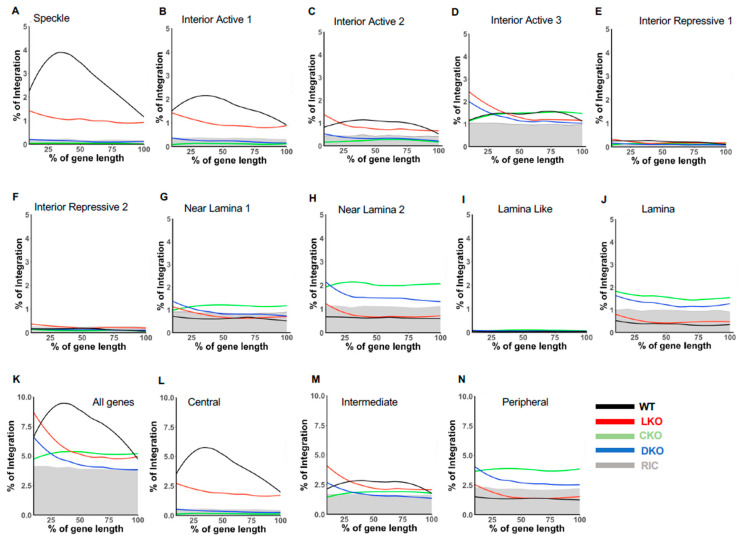
Distribution of HIV-1 integration sites as a function of gene length and SPIN state. (**A**–**J**) SPIN state genes were divided into 10 equal segments and percent integration (Y-axis) for each segment was calculated. (**K**) Total gene length integration targeting histograms for indicated HEK293T cells (WT or KO); RIC is indicated as gray shade throughout the figure. (**L**–**N**) Gene length targeting frequencies for central, intermediate, and peripheral state subgroupings. KO cells are indicated by red (LKO), green (CKO), and blue (DKO) lines.

Genic HIV-1 integration under baseline infection conditions preferentially occurs in the mid-regions of gene bodies [6,21,53]. To further investigate the granularity between spatial coordinates and genic HIV-1 integration targeting, we assessed the contributions of speckle-proximal chromatin on integration into gene mid-regions. Genes targeted for integration in HEK293T cells were segmented into 10 bins of equal length and stratified according to SPIN state. Percent integration into each bin was then quantified. The results demonstrated that genes overlapping Speckle and Interior Active 1 SPIN states displayed pronounced mid-body integration, with this preference diminishing as one moved outward from the nuclear interior (Figure 7A–J; Figure 7K shows the integration pattern across all genes).

**Figure 8:**
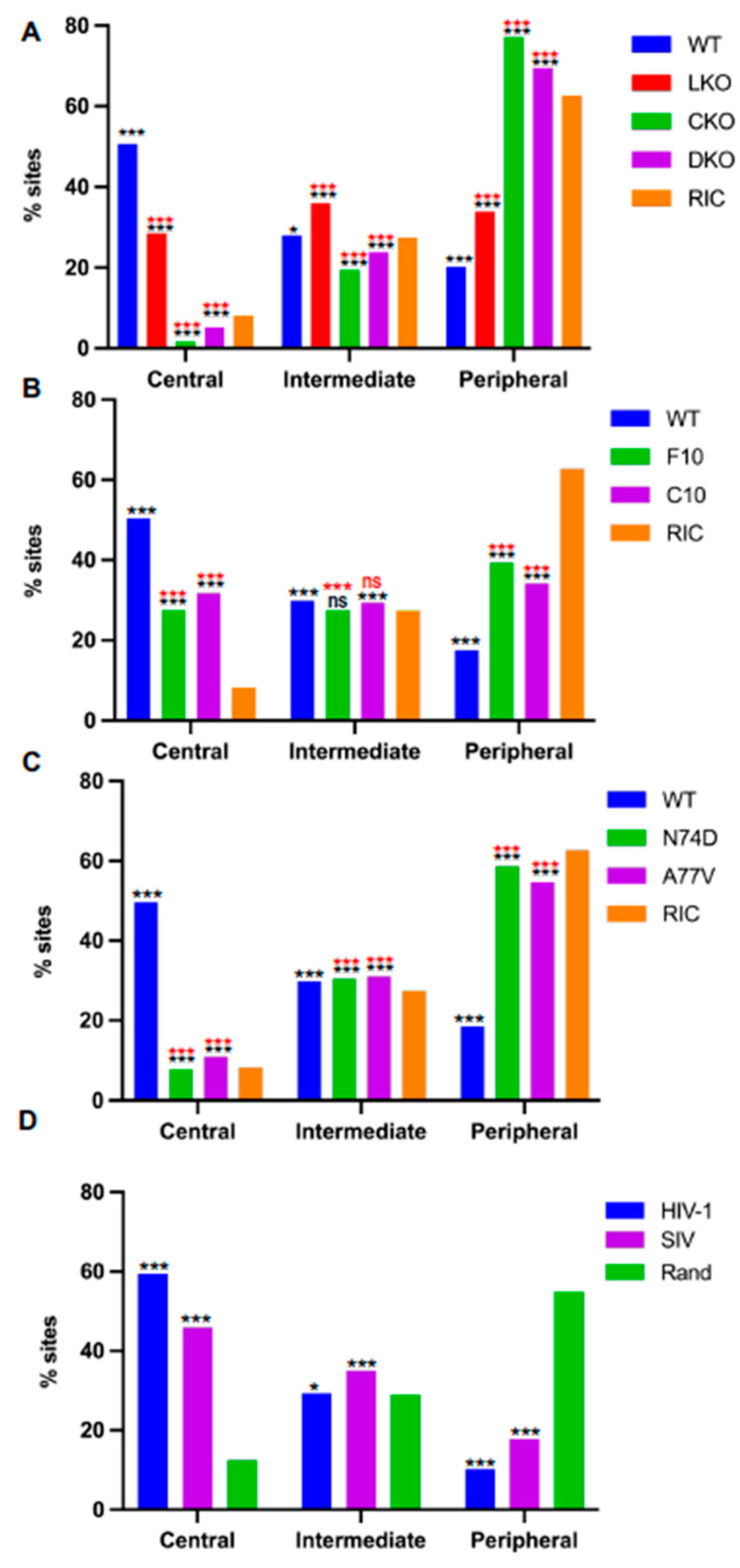
Distribution of HIV-1 integration sites into different SPIN state subgroupings. (**A**) Integration targeting frequencies in central, intermediate, and peripheral groups in WT and indicated HEK293T knockout cells (**B**) Integration targeting frequencies in WT Jurkat T cells as well as in F10 and C10 LKO cell lines. (**C**) Integration targeting frequencies in Jurkat T cells infected with WT, N74D, or A77V CA mutant virus. (**D**) HIV-1 and SIV integration targeting frequencies in human PBMCs. Orange bars, random integration control (RIC, panels **A**–**C**); green bars in panel (**D**) are random (Rand) control. *p* values, calculated using Fisher’s exact test, show differences versus RIC or Random (Rand) in black and versus WT cells in red. ***, *p* < 0.0001; *, *p* < 0.05. ns means not significant. See Tables S17–S20 for comprehensive listing of *p* values.

To facilitate interpretation across cell types, we next categorized SPIN states into three distinct groups based on mid gene-body integration targeting phenotype, which we dubbed central, intermediate, and peripheral (Figure 7L–N). In this way, Speckle and Interior Active 1 composed the central states, Interior Active 2/3 and Interior Repressive 1/2 composed the intermediate states, and the peripheral states were made up of the four outermost Lamina-related SPIN states. Depending on the methodology used to control for random integration targeting frequency [15,39], the central, intermediate, and peripheral states comprised ~10%, 28%, and 59% of the human genome, respectively (Figure 8A,D). The central states were the most highly targeted (~5- to 6-fold enriched versus random) for integration independent of cell type (Figure 8A–D; see Tables S17–S20 for corresponding *p* values). The intermediate states, by contrast, were targeted at frequencies that in large part mirrored random, while the peripheral states were disfavored for integration by as much as 5-fold compared to random (Figure 8). While LKO reduced the frequency of integration into the central states by about 20%, HIV-1 disfavored these genomic regions in CKO HEK293T cells (Figure 8A). Infection with CPSF6 binding-defective viruses similarly reduced integration targeting of central states to close to random in Jurkat T cells (Figure 8C). Reciprocally, integration into peripheral chromatin was enhanced significantly in CKO cells and in CA mutant viral-infected Jurkat T cells (Figure 8).

While integration into gene mid-regions was highly enriched in the central states, it was highly disfavored in the peripheral states independent of cell type (Figure 7L–N and Figure S11). Preferential targeting of upstream gene regions in LKO cells was seen to largely partition to the intermediate states in HEK293T and Jurkat T cells (Figure 7M and Figure S11G).

We next correlated genic integration targeting phenotypes with SPADs and regions of H3K36me3, Pol II, and LEDGF/p75 association. Mid-gene body integration targeting strongly tracked with H3K36me3- and Pol II-associated genes across cell types. While the brunt of mid-region targeting also tracked with SPAD-associated genes, SPAD-non-associated genes supported a noticeable fraction of mid-gene targeted integration (Figures S12–S15). By contrast, mid-region targeting seemed to partition fairly equally between LEDGF/p75-associated and non-associated genes.

The preference to integrate toward gene 5′ regions in LKO cells tracked with H3K36me3- and Pol II-associated genes in HEK293T and in Jurkat T cells (Figures S12D,H and S13D,H). By contrast, integration toward upstream gene regions in LKO cells predominantly tracked with SPAD-non-associated genes (Figures S12C,G and S13C,G). SPAD-non-associated genes, akin to H3K36me3- and Pol II-associated genes, supported integration targeting frequencies greater than random in CKO cells and in Jurkat T cells infected with CPSF6 binding defective CA mutant viruses (Figures S12–S14).

## 4. Discussion

HIV-1 integration has been correlated with numerous independent genetic and spatial markers of the human genome. In this study, we examined how these correlates of integration relate to one another. Our analyses informed the granularity of integration biases inherent to HIV-1 infection, as well as how host factor interactions influence these biases.

Although prior work indicated that super enhancers tracked strongly with repeated genic HIV-1 integration targets [52], we subsequently determined that this was likely due to the confluence of super enhancers with SPADs [13]. Herein, we extended meta-analytic correlates of HIV-1 integration targeting, which has highlighted specific subsets of genomic DNA that coalesce to account for the majority of previously described preferences of HIV-1 integration targeting. Gene density is a long-standing hallmark of such preferences [3] and our analyses revealed that nuclear speckle-proximal SPADs and SPIN state genomic DNA fragments are hyper-enriched for genes (Figure 1A, Table S2). Highlighting the dominant attraction of speckle-proximal chromatin, chromosomes enriched for speckle markers were preferentially targeted for integration despite otherwise similar gene densities and transcriptional profiles of less-targeted chromosomes (Figure 2, Figures S3 and S5). By comparing genic integration profiles in matched WT and LKO cells, we moreover determined that the preference for HIV-1 to target gene mid-regions in large part mapped to SPAD-associated genes (Figures S12 and S13). Our results further stratified the role played by gene density in integration targeting, as gene-dense SPAD-proximal regions of the genome were greatly preferred for primate lentiviral integration across cell types (Figure 5).

Our findings are consistent with previous results that genic targets of HIV-1 integration are generally enriched for intron content [6,21]. At the same time, we showed that intronless genes in speckle-associated and speckle-proximal chromatin were notably targeted for integration relative to spatially-matched intron-containing genes (Figure 4 and Figure S9). We speculate that this is due to the high degree of transcriptional activity common to intronless genes [21]. Although these findings seemingly contradict the prior conclusion that intron density is the staunchest predictor of HIV-1 integration site targeting, we have not conducted similar side-by-side multivariate analyses [21]. Given this limitation, we conclude that gene-dense, speckle-associated chromatin amass as the dominant predictors of HIV-1 integration targeting preferences (Figure 5). Our findings moreover suggest that pre-mRNA splicing may play a more important role in HIV-1 integration targeting of genes that do not associate with SPADs as compared to the genes that are naturally preferred for HIV-1 integration (Figure 4, Figures S8 and S9 and Table S15). Additional research is required to further test this model and, if upheld, determine the underlying molecular mechanisms.

Despite the established role of LEDGF/p75 in HIV-1 integration, genes associated with chromatin-bound LEDGF/p75 were less predictive of integration targeting as compared to H3K36me3- and Pol II-associated genes. Bedwell et al. previously speculated that the role of LEDGF/p75 in integration targeting could be more complex than static chromatin occupancy [13]. The strong correlation of both Pol II occupancy and intron number with integration site selection, along with the interaction of LEDGF/p75 with mRNA splicing factors and its role in transcription elongation, suggests potential transient interactions of LEDGF/p75 with chromatin during HIV-1 integration [21,27]. As we are aware of only one LEDGF/p75 ChIP-Seq dataset [27], the generation of orthologous LEDGF/p75 ChIP-Seq data, and potential RNA interactome datasets, will predictably further inform the mechanistic basis of LEDGF/p75 in HIV-1 integration site targeting [13].

Our results additionally clarified that, despite previous suppositions [6], integration in the absence of CPSF6 was not explicitly targeted away from gene-dense chromatin. Instead, integration simply preferred more peripheral regions, but targeted gene-dense and gene-poor chromatin within these peripheral regions similarly (Figure 6).

Our study has refined what is known about HIV-1 integration targeting biases, which we have whittled down to a select few regions/features that are highlighted by gene-dense, speckle-proximal chromatin. Future studies will be required to ascertain additional aspects of integration targeting biases, such as how these relate to proviral transcription and/or latent viral infection.

## Data Availability

The accession codes for all data analyzed in this study are provided in Supplementary Table S1.

## Supplementary Materials

The following are available online at https://www.mdpi.com/article/10.3390/cells11040655/s1, Figure S1: Distribution of size-matched random fragments with respect to gene density; Figure S2: SPIN state distributions with respect to gene density; Figure S3: SPIN sate distributions among human chromosomes; Figure S4: Distributions of random sites per chromosome; Figure S5: Chromosomal distributions of retroviral integration sites; Figure S6: HIV-1 integration targeting frequencies in different sets of Jurkat T cell genes; Figure S7: WT and CA mutant HIV-1 integration profiles in different gene sets; Figure S8: HIV-1 and SIV integration profiles in different human PBMC gene sets; Figure S9: Primate lentiviruses preferentially target speckle-associated intronless genes for integration in T cells; Figure S10: Integration targeting frequencies using previously determined methodology [21] as a function of intron content; Figure S11: Lengthwise distributions of genic HIV-1/SIV integration targeting in T cells; Figure S12: Integration targeting of specific gene subsets in WT and knockout HEK293T cells; Figure S13: Integration targeting of specific gene subsets in WT and LKO Jurkat T cells; Figure S14: Integration targeting of specific gene subsets in Jurkat T cells; Figure S15: HIV-1/SIV integration targeting of specific gene subsets in PBMCs; Table S1: Accession numbers and data sources; Table S2: Gene densities of genomic annotations; Table S3: Gene density *p* values versus random; Table S4: Overlap of SPADs with SPIN states; Table S5: Average gene densities of human chromosomes; Table S6: Pearson correlation analyses at the chromosomal level; Table S7: Human chromosome enrichments versus random; Table S8: HIV-1 integration enrichments per chromosome; Table S9: Statistical analysis of HIV-1 targeting frequencies in HEK293T cells; Table S10: Statistical analysis of HIV-1 targeting frequencies in WT and LKO Jurkat T cells; Table S11: Statistical analysis of WT and CA mutant HIV-1 integration targeting frequencies in Jurkat T cells; Table S12: Statistical analyses of HIV-1 and SIV integration frequencies in PBMCs; Table S13: Intron content differences across gene type; Table S14: Differences in integration targeting frequencies in intronless versus intron-containing genes; Table S15: Correlation between intron group and integration; Table S16: SPIN state integration targeting stratified by gene density; Table S17: HIV-1 integration enrichment as a function of SPIN state in WT and KO HEK293T cells; Table S18: HIV-1 integration enrichment as a function of SPIN state in WT and LKO Jurkat T cells; Table S19: HIV-1 integration enrichment in SPIN subgroups in WT versus CA mutant infected Jurkat cells; Table S20: HIV-1 and SIV enrichment in SPIN subgroups in PBMCs.
