# Supplementary material for: Spatial and Genomic Correlates of HIV-1 Integration Site Targeting
Source: Cells. 2022 Feb 14;11(4):655. doi: 10.3390/cells11040655 (PMC8869898; doi:10.3390/cells11040655)
Supplement: Supplementary file 1 [file cells-11-00655-s001.zip › cells-1590222-supplementary/cells-1590222 for conversion sm final/cells-1590222-supplementary-version2/cells-1590222 supplementary figures_version2.pdf]

cells

MDPI

Supplementary Materials

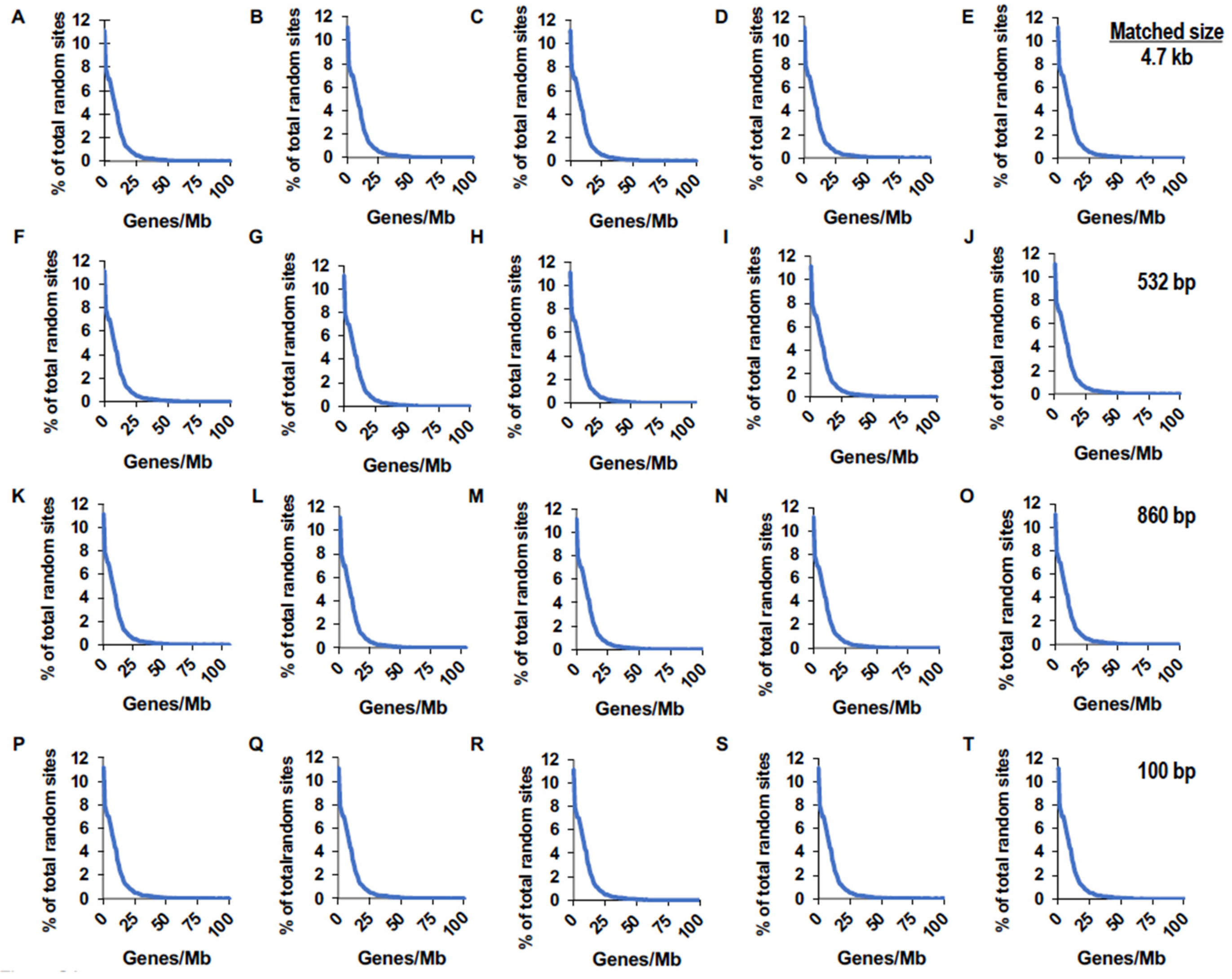

Figure S1. Distribution of size-matched random fragments with respect to gene density. Fragments were selected computationally to match average sizes of ChIP-Seq DNA fragments for H3K36me3 (**A–E**; 4.7 kb), LEDGF/p75 (**F–J**; 532 bp), and Pol II (**K–O**; 860 bp); panels **P–T** matched the average size of SPAD fragments (100 bp). For each matched-size group, five independent sets of 1,000,000 fragments each were analyzed, except for the 100 bp group (panels P−T), where each analysis contained 1,547,458 fragments. For each random fragment, average gene density (X-axis) was calculated in a 1 Mb genomic region (+/− 500 kb from the fragment midpoint). Y-axes show the % of sites having the calculated gene density.

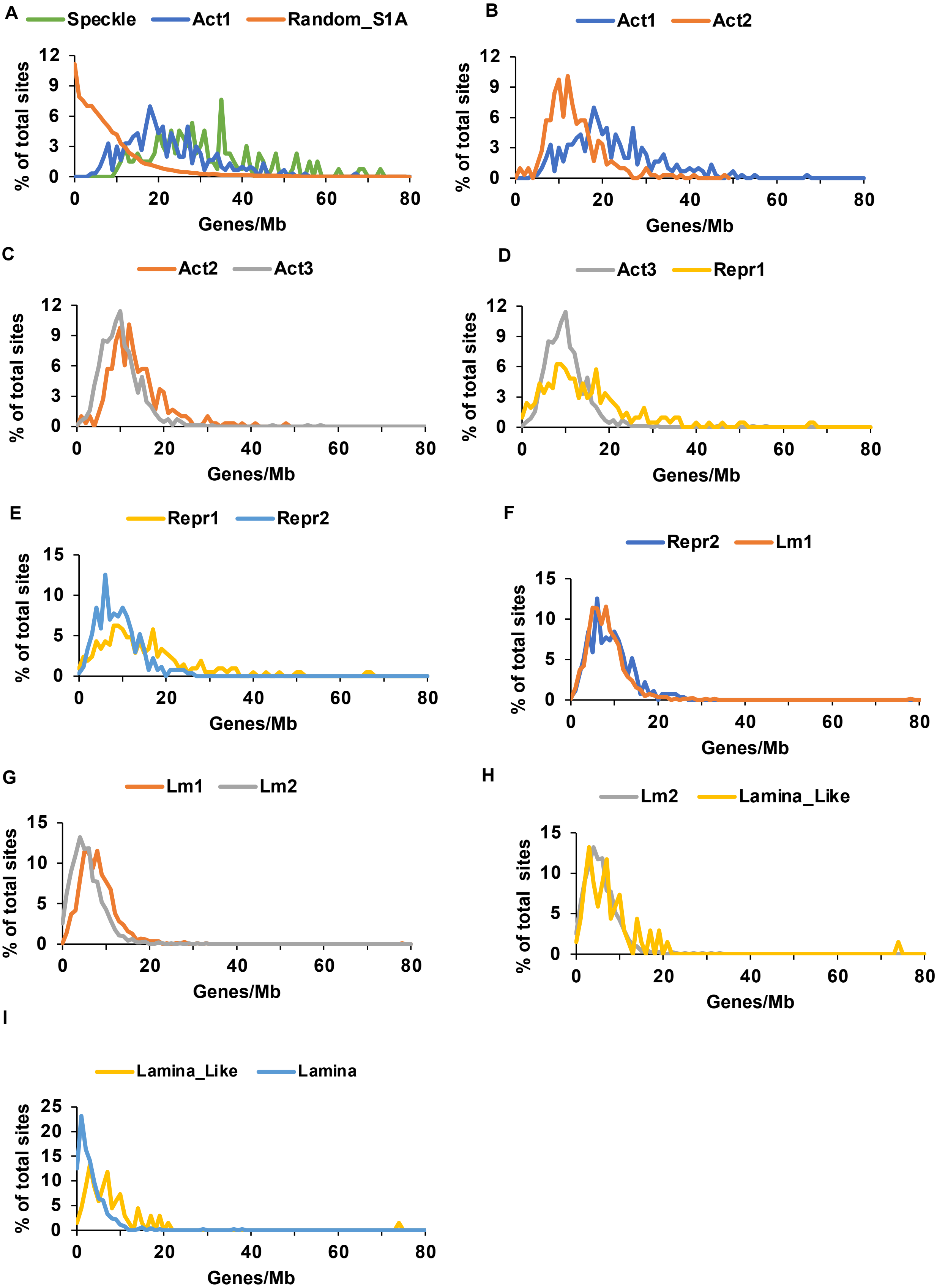

Figure S2. SPIN state distributions with respect to gene density. (**A–I**) For each SPIN site, average gene density (X-axis), which was calculated in 1 Mb regions (+/− 500 kb from midpoint site), was plotted versus % of sites of a SPIN state having the same gene density (Y-axis). For clarity, SPIN states were plotted in progressive pairwise fashion from the nuclear interior in panel A to the periphery in panel I. The random curve generated in Figure S1A is replotted in panel A. SPIN

state abbreviations: Act1, Interior Active 1; Act2, Interior Active 2; Act 3, Interior Active 3; Repr1, Interior Repressive 1; Repr2, Interior Repressive 2; Lm1, Near Lamina 1; Lm2, Near Lamina 2.

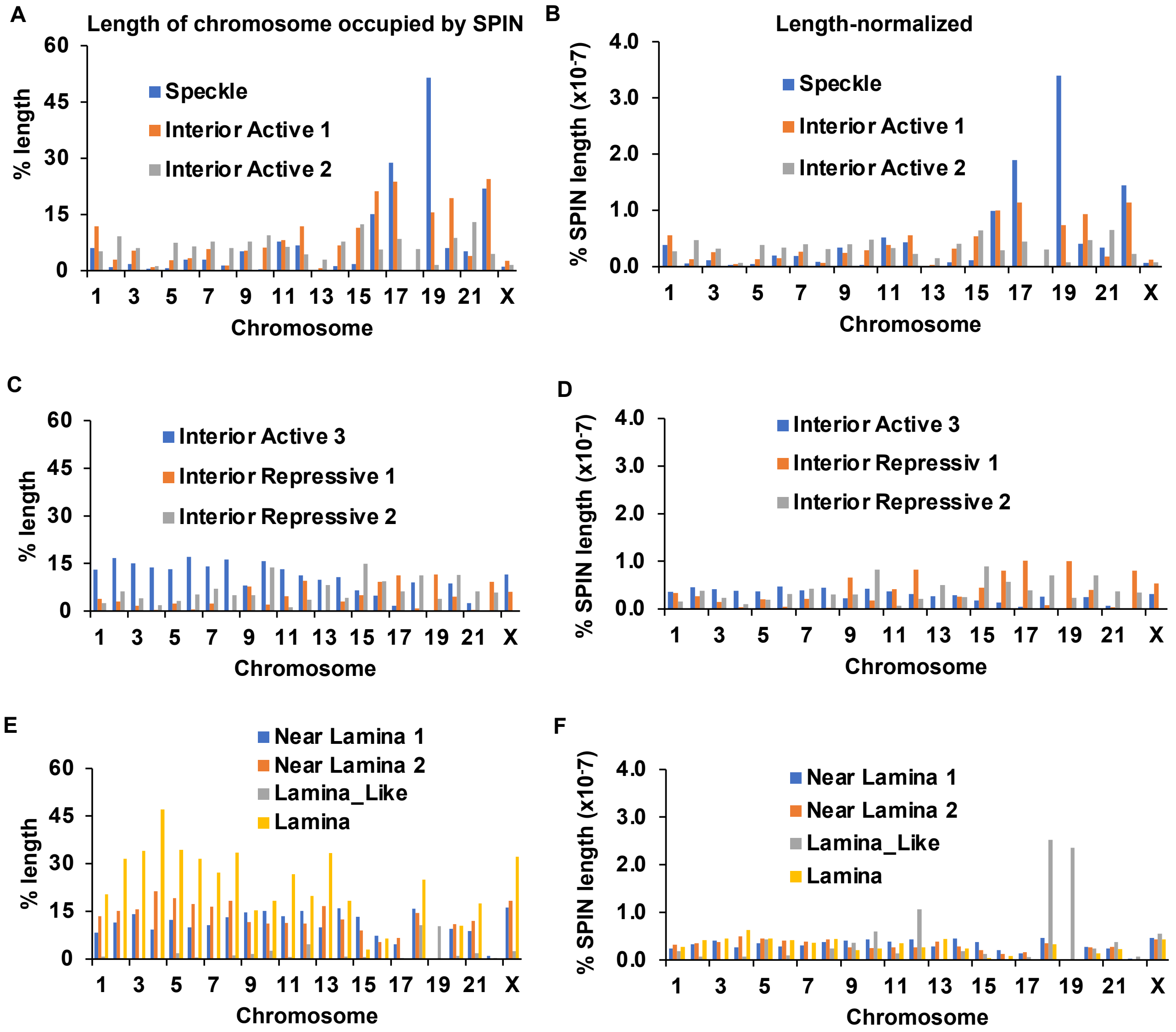

Figure S3. SPIN sate distributions among human chromosomes. (**A, C and E**) The total length of each indicated SPIN state was expressed as % length of the corresponding chromosome (Y-axis) (**B, D and F**) Corresponding panel A, C and E data normalized by the total length of each SPIN state, which was calculated by adding the lengths of the indicated SPIN state fragments.

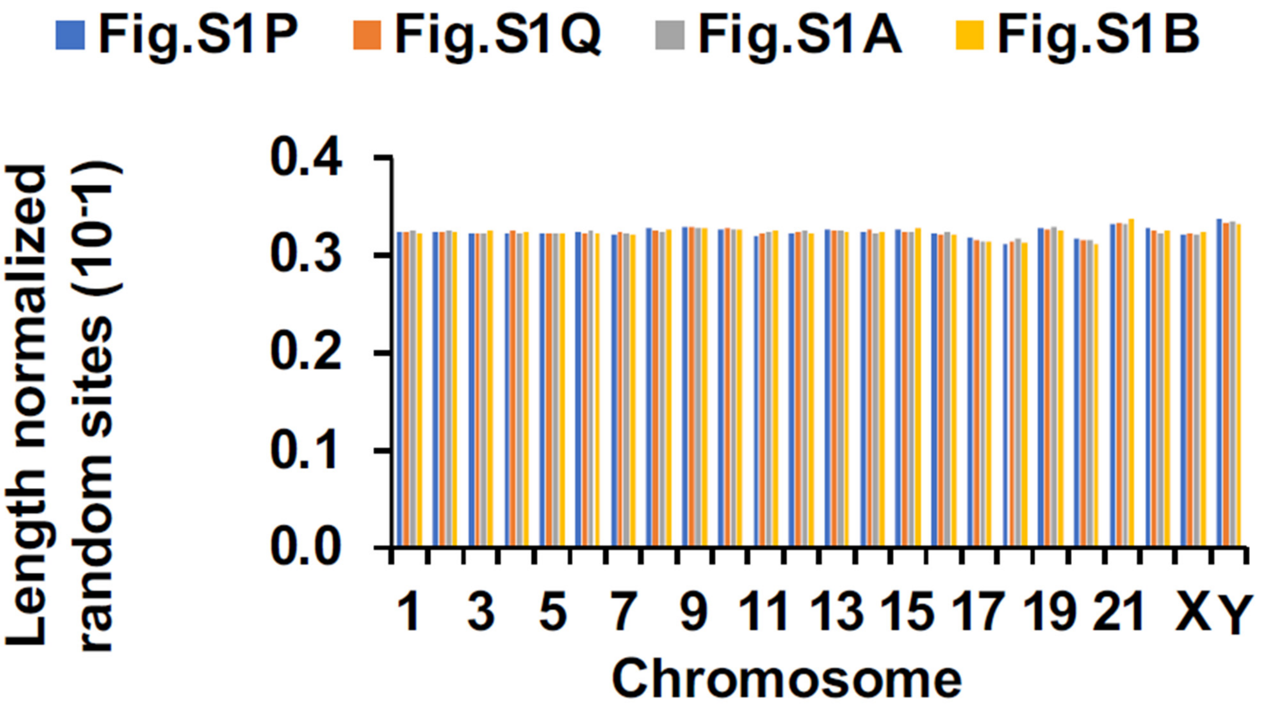

Figure S4. Distributions of random sites per chromosome. For each chromosome, random sites from indicated Figure S1 histograms were counted (%) and normalized to the length of the indicated chromosome. The Y-axis shows the % of random sites /Mb.

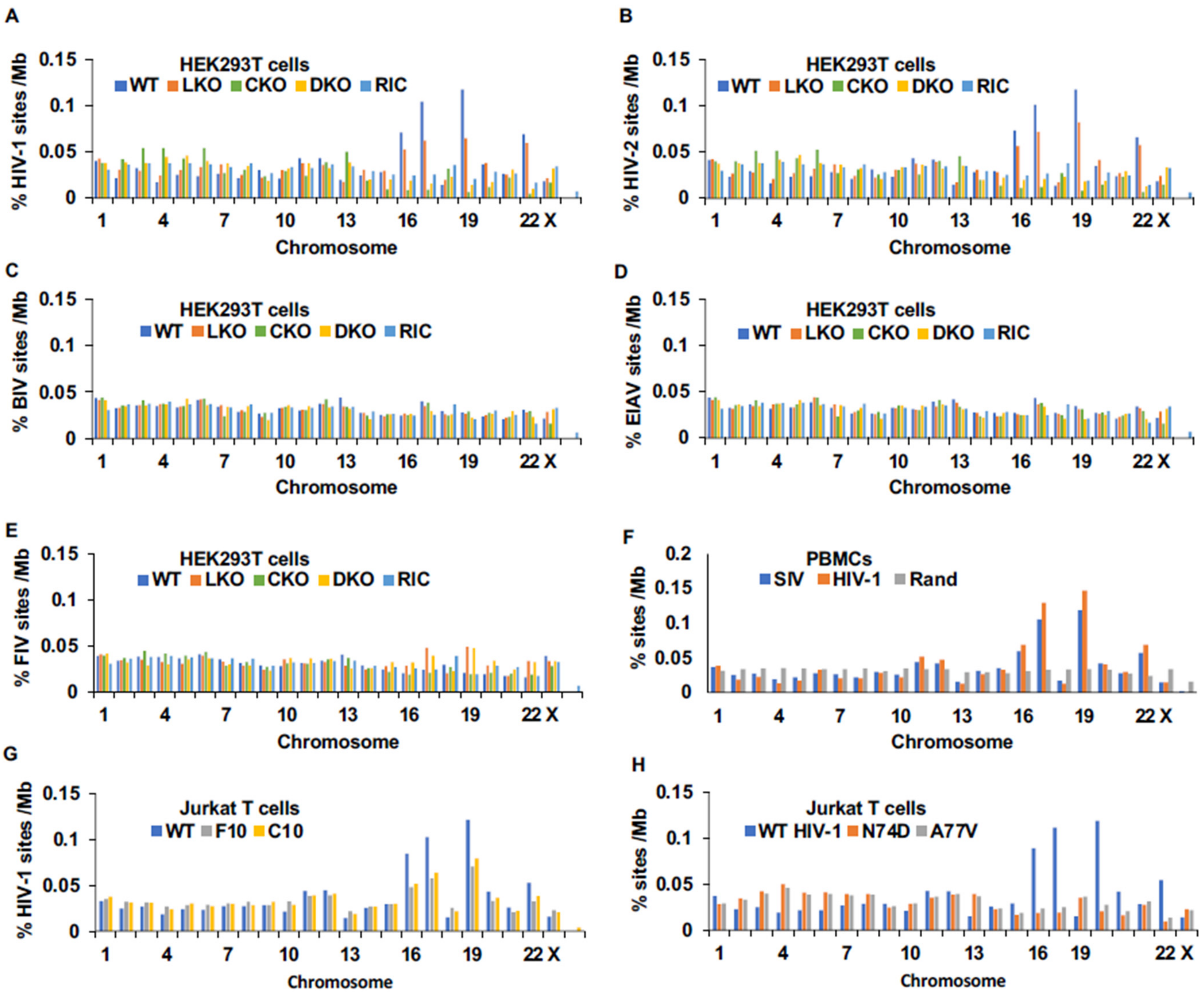

Figure S5. Chromosomal distributions of retroviral integration sites. (**A**) HIV-1 integration site distributions for indicated HEK293T cell type. (**B-E**) As in panel A, for HIV-2 (B), BIV (C), EIAV (D) and FIV (E) integration sites. (**F**) Distributions of HIV-1 and SIV integration sites in human PBMC. (**G**) HIV-1 integration site distributions in WT Jurkat versus two different LKO cell lines (F10 and C10). (**H**) Integration site distributions in Jurkat T cells infected with WT versus CA mutant N74D or A77V HIV-1. Y-axes are percent integration sites normalized to chromosome length in Mb. Study-matched random integration control (RIC) and Random (Rand) distributions are indicated. The RIC from panel A also applies to panels G and H.

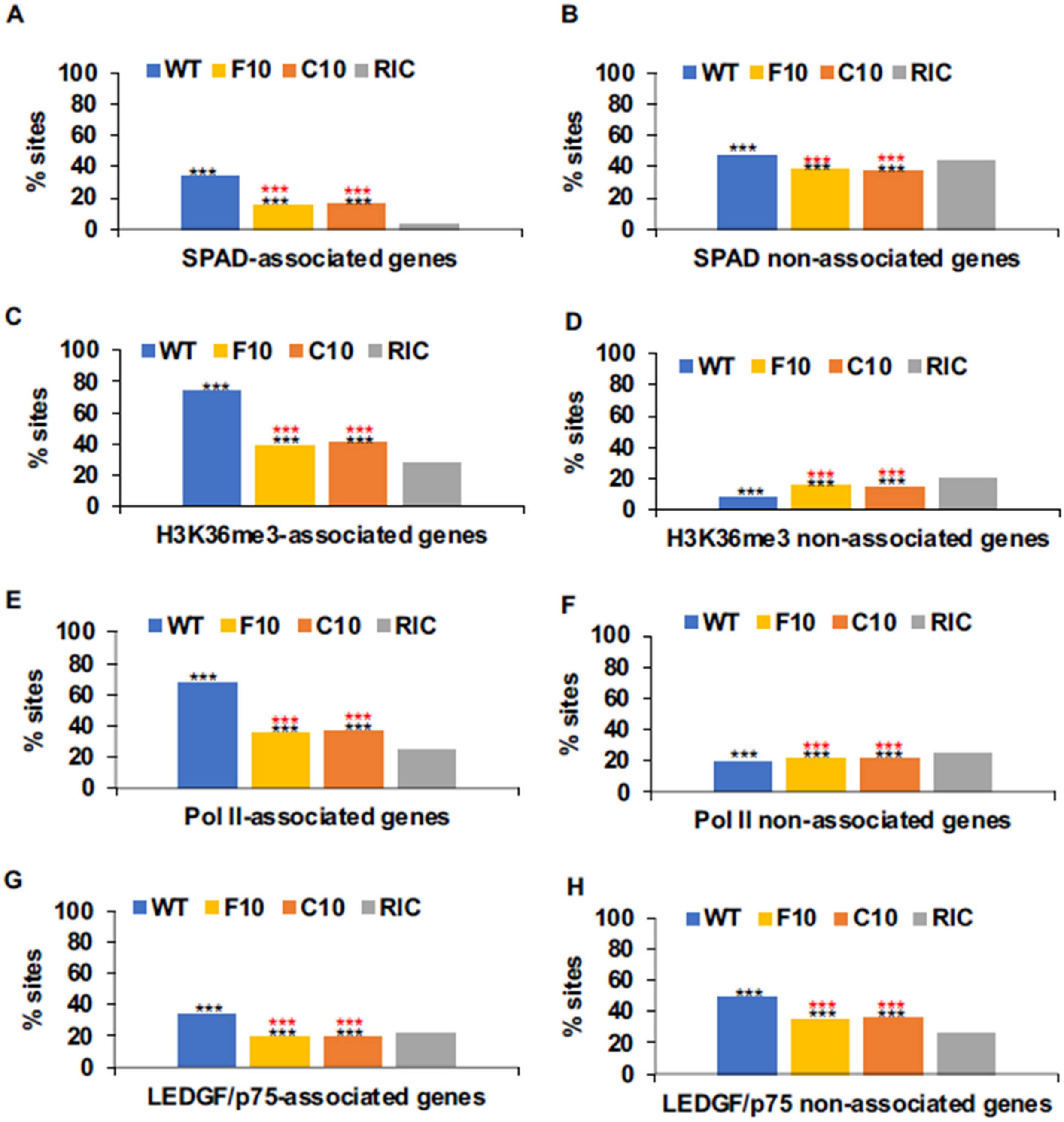

Figure S6. HIV-1 integration targeting frequencies in different sets of Jurkat T cell genes. (**A-B**) Integration into SPAD-associated (panel A) and non-associated (panel B) genes for the indicated Jurkat T cell type; F10 and C10 are two different LKO cell lines. (**C-D**) Integration into H3K36me3-associated (panel C) and non-associated (panel D) genes. (**E-F**) Integration into Pol II-associated (panel E) and non-associated (panel F) genes. (**G-H**) Integration into LEDGF/p75-associated (panel G) and non-associated (panel H) genes. RIC, random integration control. P values, calculated using Fisher's exact test, show differences versus RIC in black and versus WT Jurkat T cells in red (***, $P < 0.001$). See Table S10 for comprehensive listing of P values.

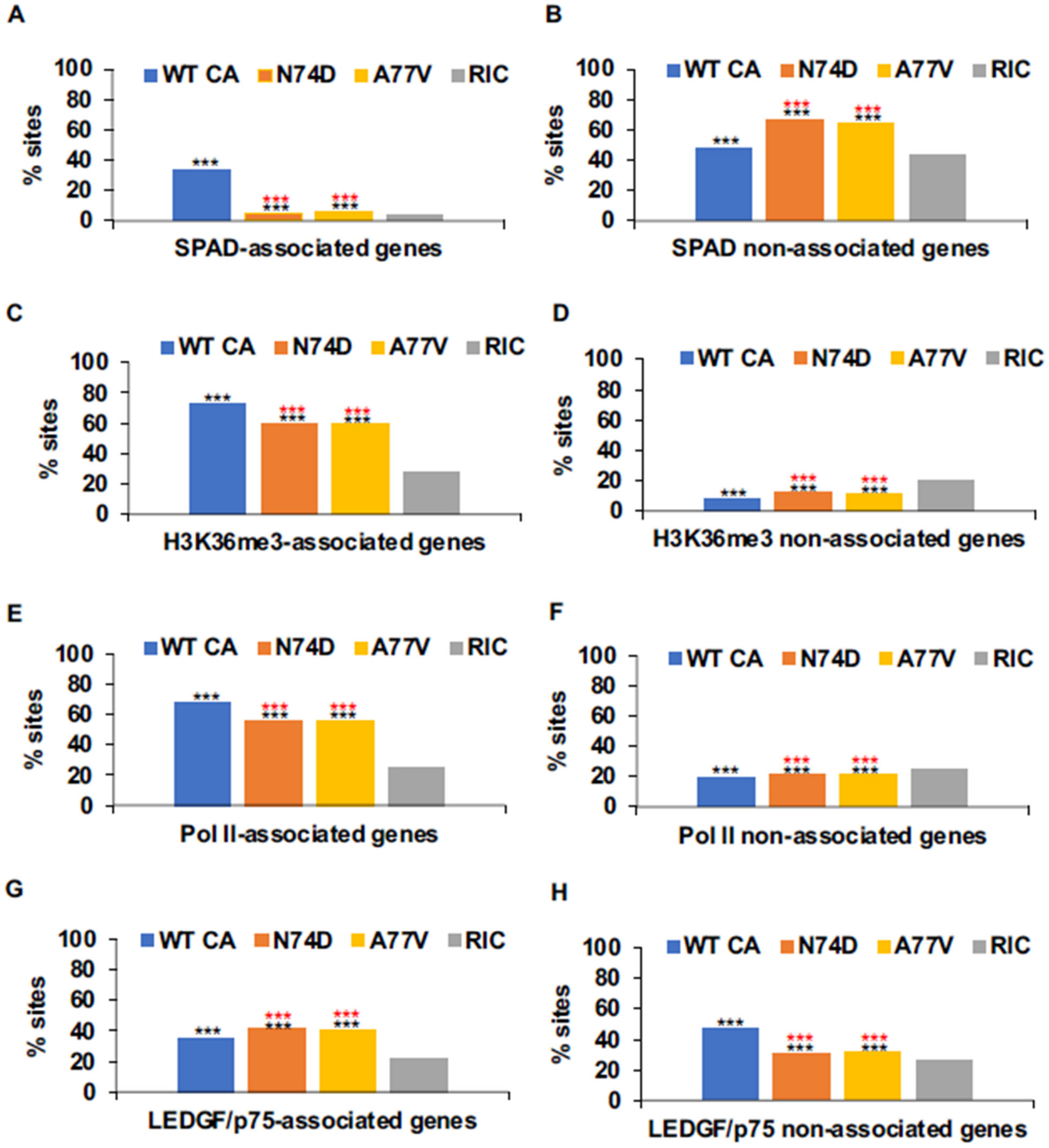

Figure S7. WT and CA mutant HIV-1 integration profiles in different gene sets. (**A-B**) Integration into SPAD-associated (panel A) and non-associated (panel B) genes for the indicated virus. (**C-D**) Integration into H3K36me3-associated (panel C) and non-associated (panel D) genes. (**E-F**) Integration into Pol II-associated (panel E) and non-associated (panel F) genes. (**G-H**) Integration into LEDGF/p75-associated (panel G) and non-associated (panel H) genes. RIC, random integration control. P values, calculated using Fisher's exact test, show differences versus RIC in black and versus WT HIV-1 in red (***, $P < 0.001$). See Table S11 for comprehensive listing of P values.

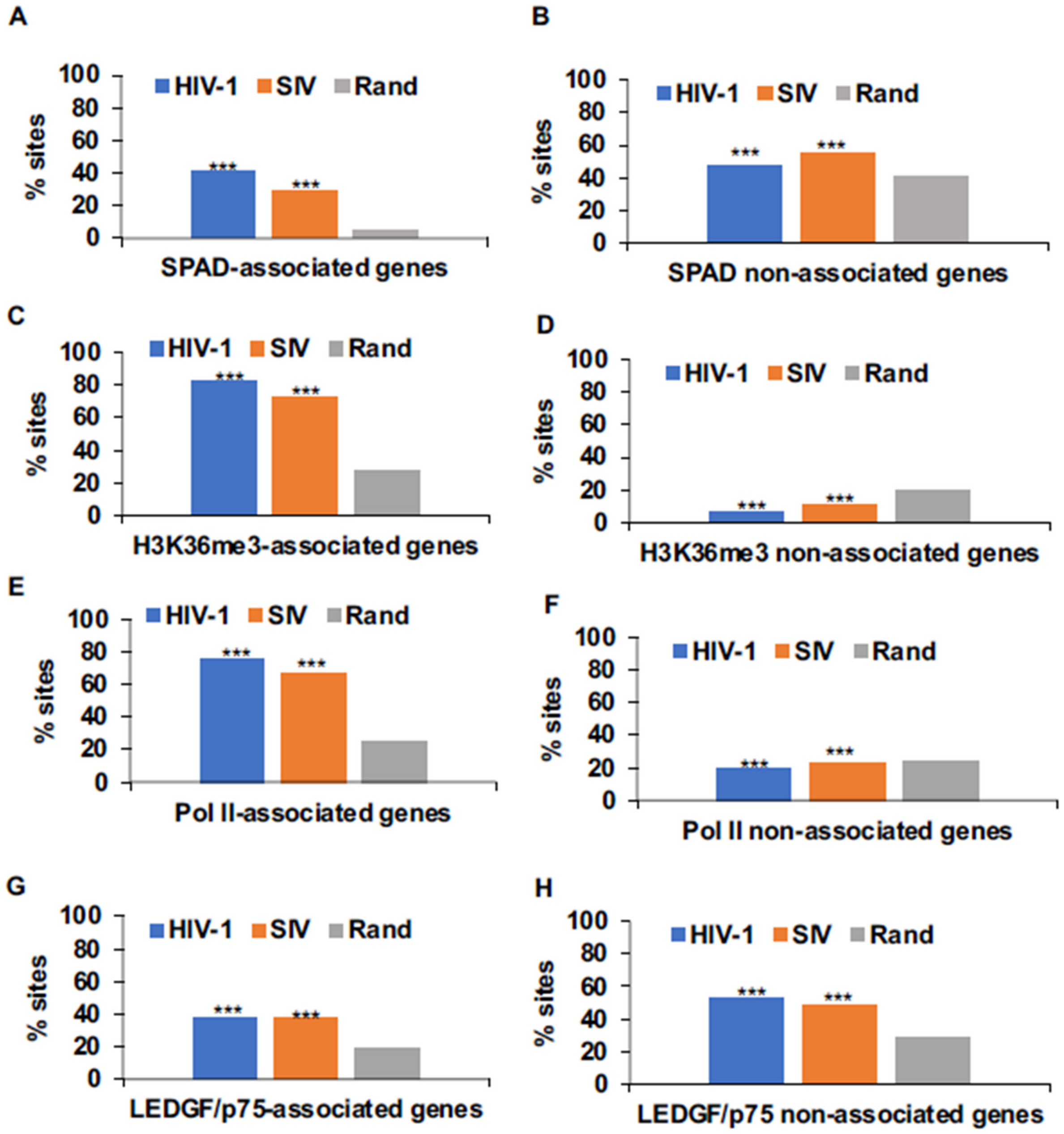

Figure S8. HIV-1 and SIV integration profiles in different human PBMC gene sets. (**A-B**) Integration into SPAD-associated (panel A) and non-associated (panel B) genes. (**C-D**) Integration into H3K36me3-associated (panel C) and non-associated (panel D) genes. (**E-F**) Integration into Pol II-associated (panel E) and non-associated (panel F) genes. (**G-H**) Integration into LEDGF/p75-associated (panel G) and non-associated (panel H) genes. Rand, random control values. P values, calculated using Fisher's exact test, show differences versus Rand (***, $P < 0.001$). See Table S12 for comprehensive listing of P values.

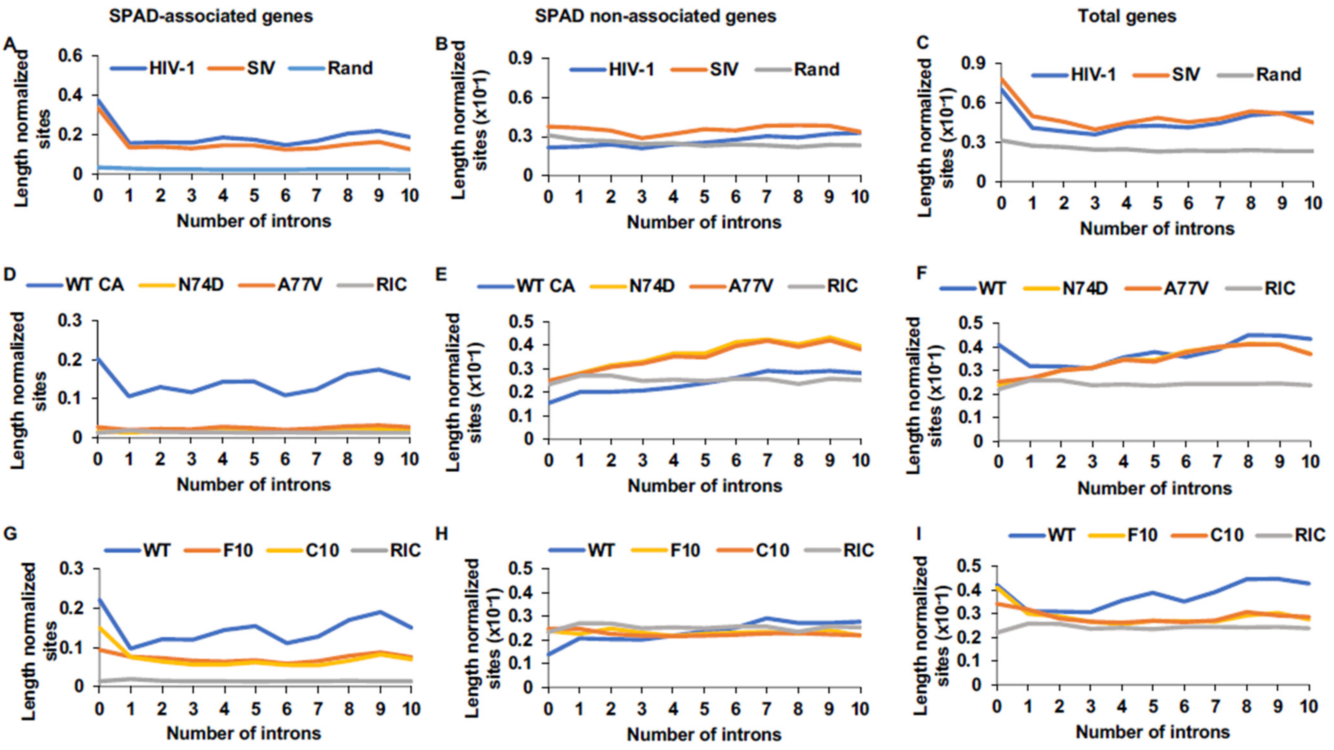

Figure S9. Primate lentiviruses preferentially target speckle-associated intronless genes for integration in T cells. (**A**) HIV-1 and SIV integration targeting frequencies in SPAD-associated genes in PBMCs as a function of intron content. Results are normalized to gene length in Mb. (**B and C**) Same as in panel A, except for SPAD non-associated genes (panel B) and total genes (panel C). (**D**) Integration targeting frequency of WT and indicated CA mutant viruses in SPAD-associated genes in Jurkat T cells as a function of intron content. Results are normalized to total gene length in Mb. (**E and F**) Same as in panel D, except for SPAD non-associated genes (panel E) and total genes (panel F). (**G**) Integration targeting frequency in SPAD-associated genes in WT and LKO Jurkat T cells as a function of intron content. Results were normalized to gene length in Mb. (**H and I**) Same as in panel G, except for SPAD non-associated genes (panel H) and total genes (panel I).

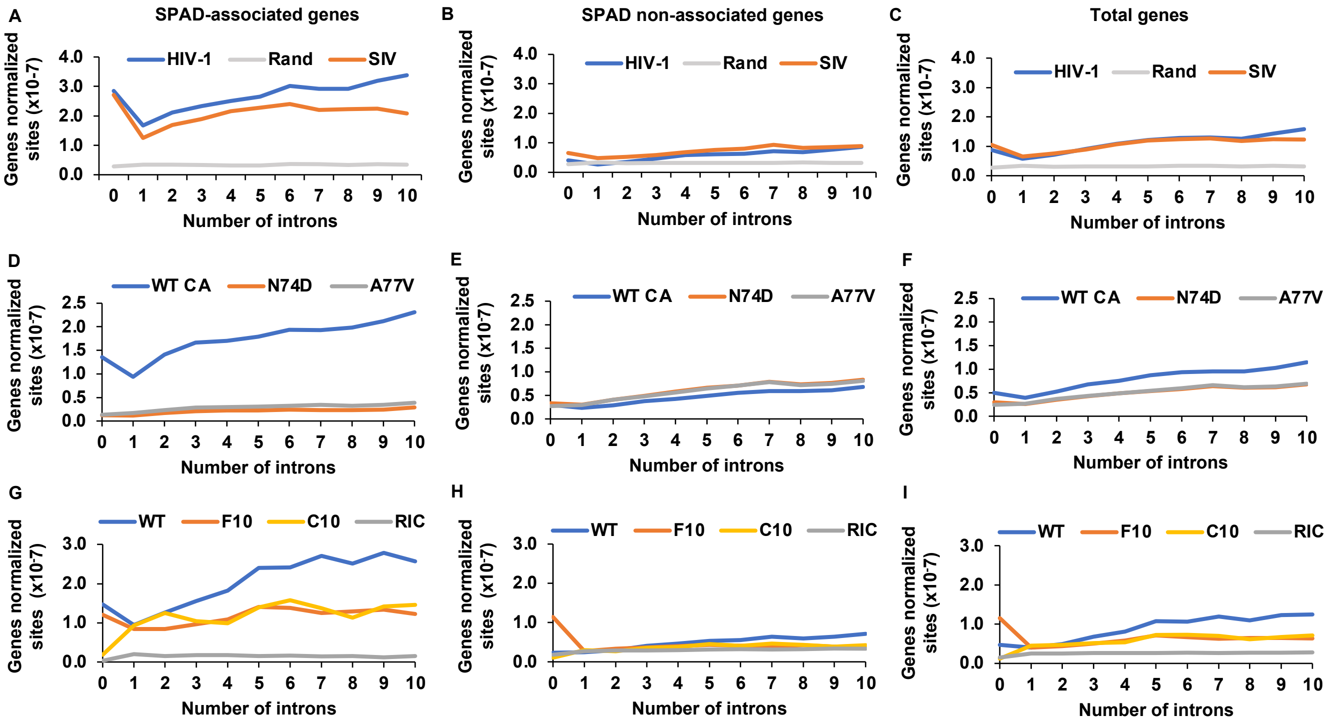

Figure S10. Integration targeting frequencies using previously determined methodology [21] as a function of intron content. (**A**) HIV-1 and SIV integration targeting frequencies in SPAD-associated genes in PBMCs as a function of intron content. (**B and C**) Same as in panel A, except for SPAD non-associated genes (panel B) and total genes (panel C). (**D**) Integration targeting frequency of WT and indicated CA mutant viruses in SPAD-associated genes in Jurkat T cells as a function of intron content. (**E and F**) Same as in panel D, except for SPAD non-associated genes (panel E) and total genes (panel F). (**G**) Integration targeting frequency in SPAD-associated genes in WT and LKO Jurkat T cells as a function of intron content. (**H and I**) Same as in panel G, except for SPAD non-associated genes (panel H) and total genes (panel I).

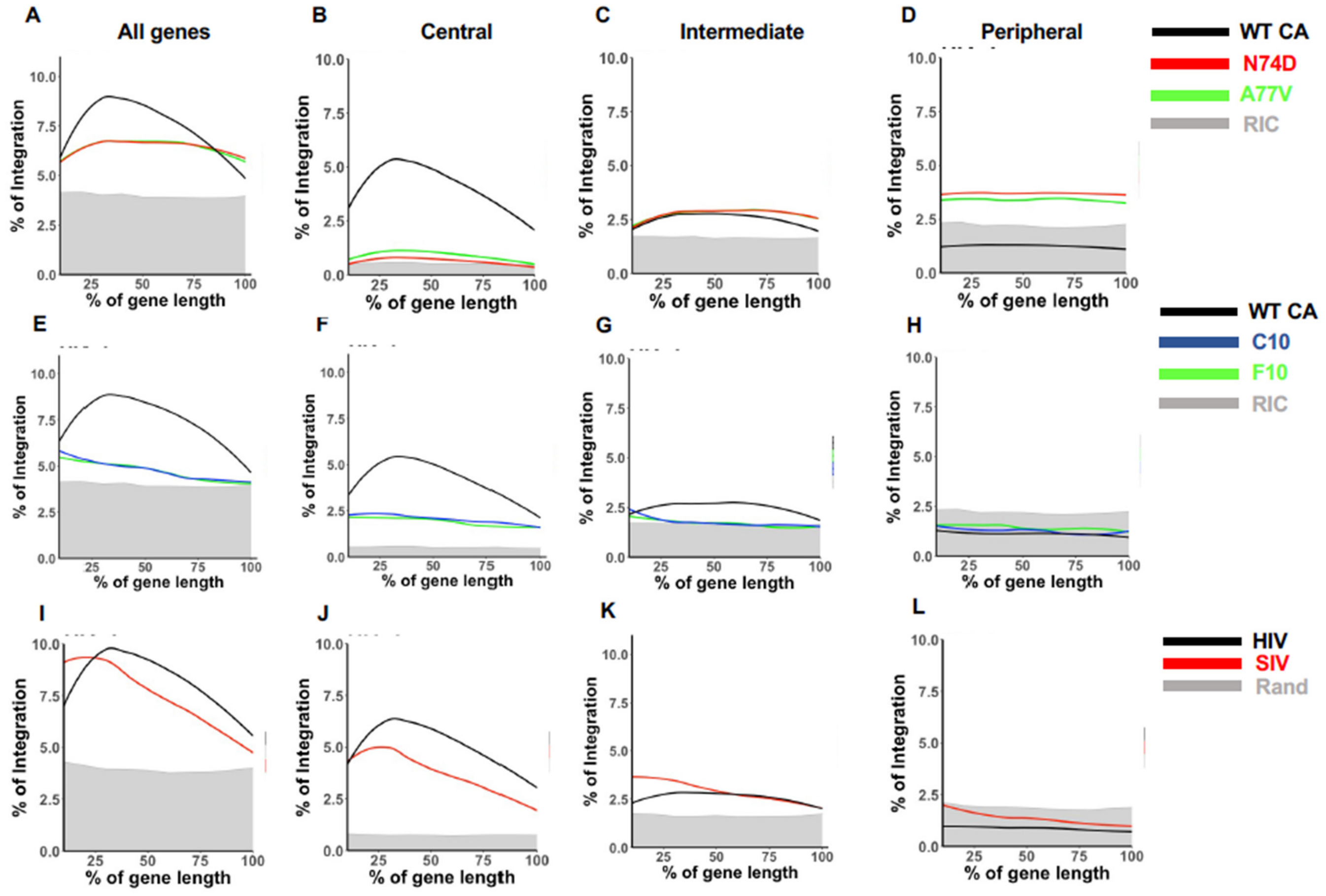

Figure S11. Lengthwise distributions of genic HIV-1/SIV integration targeting in T cells. (**A**) Genic integration targeting histograms for WT and CA mutant viral integration in Jurkat T cells. These integration sites were stratified in panels (**B-D**) as Central (B), Intermediate (C), and Peripheral (D) SPIN state subgroups. (**E**) Genic HIV-1 integration targeting curves in WT and LKO Jurkat T cell lines. Panels (**F-H**) display these data as Central (F), Intermediate (G), and Peripheral (H) SPIN state subgroups. (**I**) Genic integration profiles of HIV-1 and SIV in human PBMCs. Panels (**J-L**) stratify these data into Central (J), Intermediate (K) and Peripheral (L) SPIN state groups. Gray shade, RIC (panels A-H) and Rand (panels I-L).

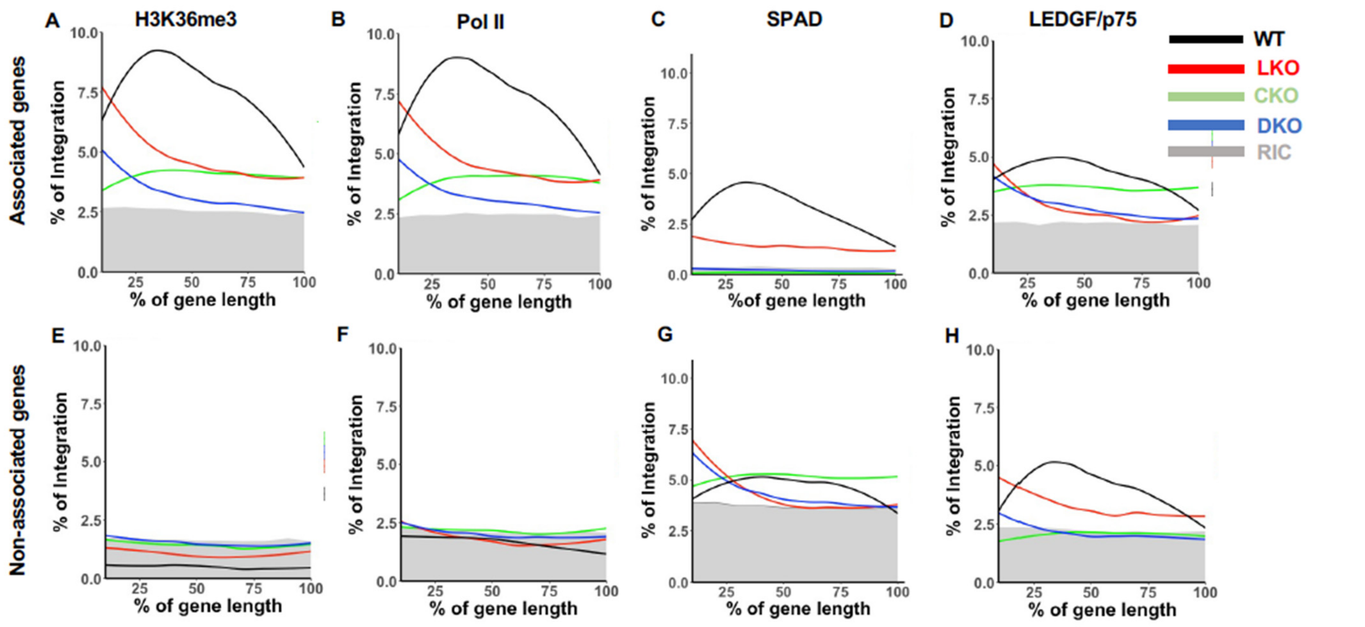

Figure S12. Integration targeting of specific gene subsets in WT and knockout HEK293T cells. (**A**) Distribution of HIV-1 integration sites across length-normalized H3K36me3-associated genes. WT, LKO, CKO, and DKO cells are indicated by black, red, green, and blue lines, respectively; gray shade indicates RIC throughout the figure. (**B-D**) Same as in A, except for Pol II-associated (B), SPAD-associated (C), and LEDGF/p75-associated (D) genes. (**E-H**) Same as in panels A-D, except for the corresponding non-associated genes.

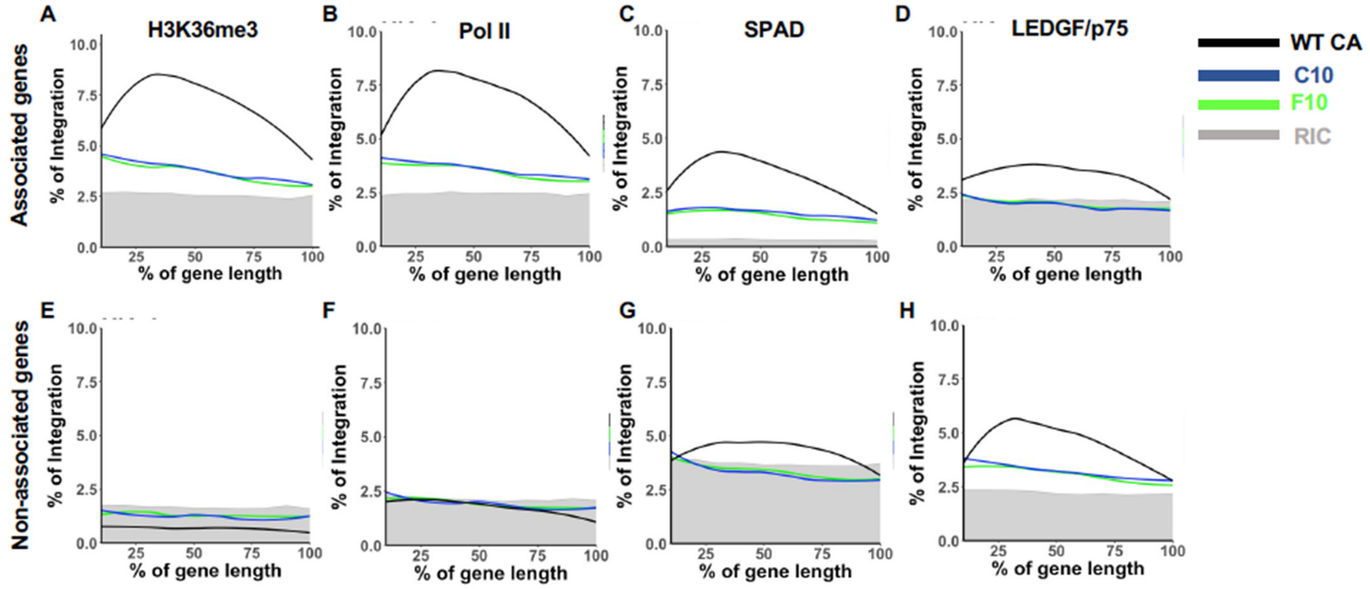

Figure S13. Integration targeting of specific gene subsets in WT and LKO Jurkat T cells. (**A**) Distribution of HIV-1 integration sites length-normalized for H3K36me3-associated genes in WT (black), C10 LKO (blue), and F10 LKO (green) Jurkat T cells. (**B-D**) Same as in A, except for Pol II-associated (B), SPAD-associated (C), and LEDGF/p75-associated (D) genes. (**E-H**) Same as in panels A-D, except for the corresponding non-associated genes. Gray shade indicates RIC throughout the figure.

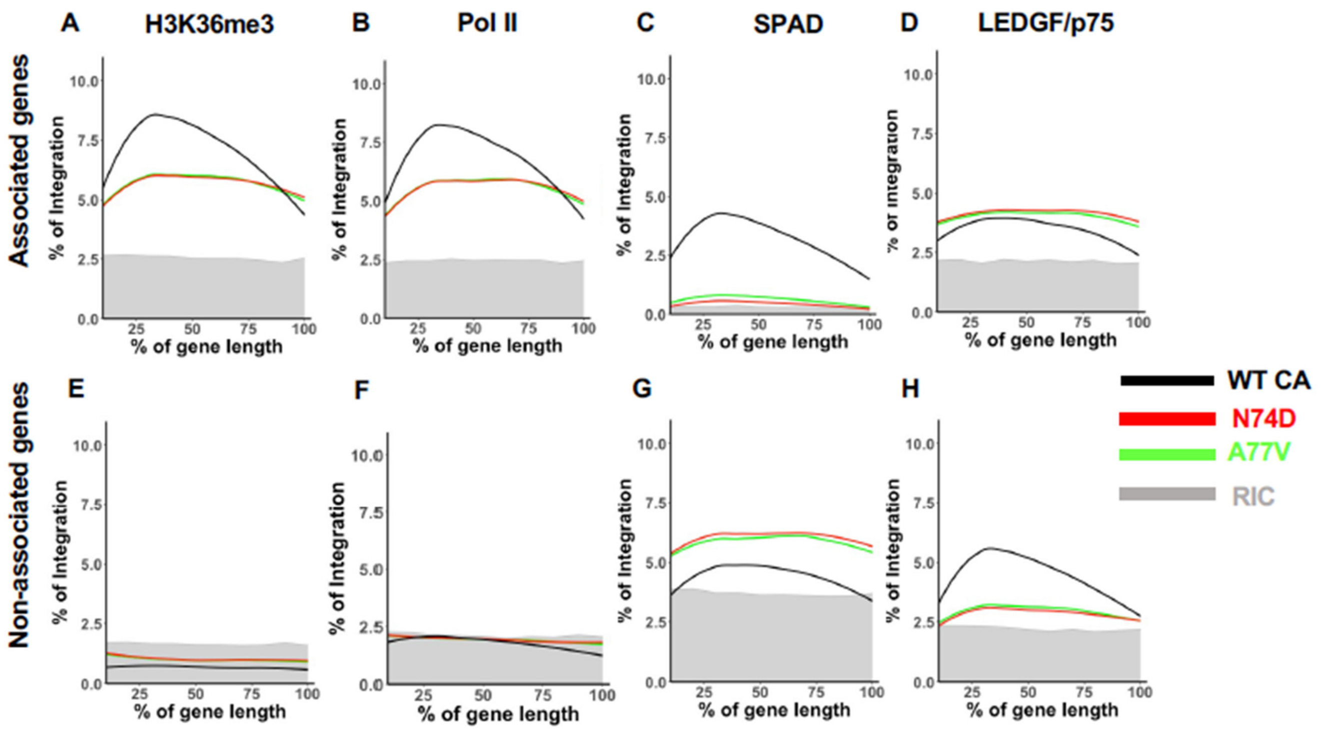

Figure S14. Integration targeting of specific gene subsets in Jurkat T cells. (**A**) Distribution of WT (black) and N74D (red) and A77V (green) CA mutant viral integration sites across length-normalized H3K36me3-associated genes. (**B-D**) Same as in A, except for Pol II-associated (B), SPAD-associated (C), and LEDGF/p75-associated (D) genes. (**E-H**) Same as in panels A-D, except for the corresponding non-associated genes. Gray shade indicates RIC throughout the figure.

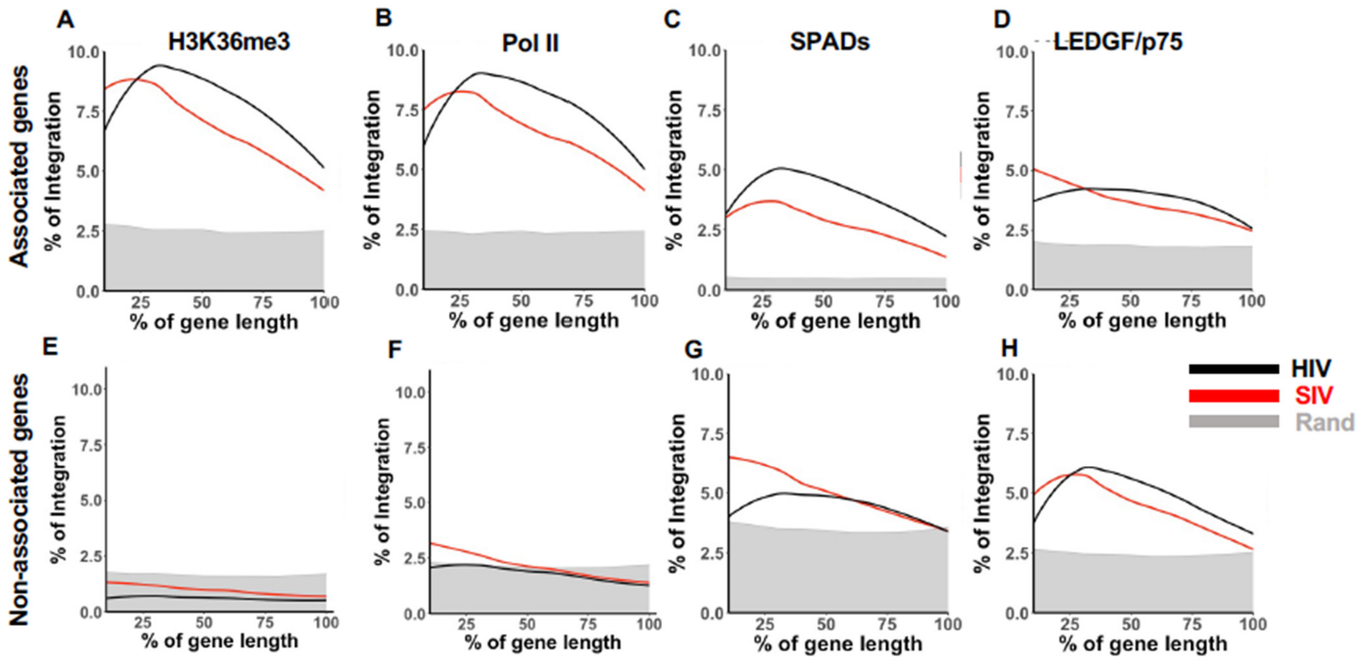

Figure S15. HIV-1/SIV integration targeting of specific gene subsets in PBMCs. (**A**) Distribution of HIV-1 (black) and SIV (red) integration sites across length-normalized H3K36me3-associated genes. (**B-D**) Same as in A, except for Pol II-associated (B), SPAD-associated (C), and LEDGF/p75-associated (D) genes. (**E-H**) Same as in panels A-D, except for the corresponding non-associated genes. Gray shade indicates Rand throughout the figure.
